# *Rv2629* Overexpression Delays *Mycobacterium smegmatis* and *Mycobacteria tuberculosis* Entry into Log-Phase and Increases Pathogenicity *of Mycobacterium smegmatis* in Mice

**DOI:** 10.3389/fmicb.2017.02231

**Published:** 2017-11-15

**Authors:** Dan Liu, Kewei Hao, Wenjie Wang, Chao Peng, Yue Dai, Ruiliang Jin, Wenxi Xu, Lei He, Hongyan Wang, Honghai Wang, Lu Zhang, Qingzhong Wang

**Affiliations:** ^1^Department of Immunology and Pathogen Biology, Shanghai University of Traditional Chinese Medicine, Shanghai, China; ^2^State Key Laboratory of Genetic Engineering, Institute of Genetics, School of Life Sciences, Fudan University, Shanghai, China; ^3^Shanghai Pulmonary Hospital, Tongji University School of Medicine, Shanghai, China; ^4^Shanghai Centre for Clinical Laboratory, Shanghai, China

**Keywords:** *Rv2629*, *Mycobacterium smegmatis*, Mycobacterium tuberculosis, bacterial physiology, dormancy

## Abstract

**Objective:** The aim of the present study was to explore the potential biological role of Rv2629 in *Mycobacterium smegmatis* and *Mycobacterium tuberculosis.*

**Methods:** Recombinant wild type and mutant *Rv2629* strains were constructed. *Rv2629* expression was evaluated by real-time PCR and western blot. Microarray and interaction network analyses were used to identify the gene interactions associated with wild type and mutant *Rv2629*. Bacterial growth was assessed in Balb/c mice infected with wild type and mutant *Rv2629* strains using CFU assay and histological analysis of the organs.

**Results:** Overexpression of *Rv2629* could delay the entry of the *Mycobacterium tuberculosis* cells into the log-phase, while *Rv2629* decreased the number of ribosomes and the expression of uridylate kinase in *Mycobacterium smegmatis*. The Gene Ontology (GO) and pathway analysis indicated that 122 genes correlated with wild type *Rv2629*, whereas the *Rv2629* mutation led to decrease in the ribosome production, oxidative phosphorylation, and virulence in *Mycobacterium tuberculosis*. Overexpression of *Rv2629* slightly enhanced the drug resistance of *Mycobacterium smegmatis* to antibiotics, and increased its survival and pathogenicity in Balb/c mice.

**Conclusion:** It is suggested that *Rv2629* is involved in the survival of the clinical drug-resistant strain via bacterial growth repression and bacterial persistence induction.

## Introduction

*Mycobacterium tuberculosis* (*M. tuberculosis*) infection is the major cause of TB. In 2016, 9287 new TB cases were reported in the United States and the successful elimination of TB in the United States is not expected to be achieved during this century (Doosti-Irani et al., 2016). The successful survival and evolution of *M. tuberculosis* is attributed to a large extent to its ability to persist for long periods within the human body in a latent and/or dormant state (Peddireddy et al., 2017). During latent infection, *M. tuberculosis* bacilli are retained within granulomas, which offers a niche with increased concentration of nitric oxide, low oxygen, and absence of nutrients (Bartek et al., 2009; Trauner et al., 2012). The non-replicating persistent form of *M. tuberculosis* aids the escape of the pathogen from the host defense mechanisms and provides additional advantage to the bacilli as regards the effect of standard anti-TB drugs. This process results in the long term survival of the bacteria for several decades (Voskuil et al., 2003; Keshari et al., 2017).

Genes such as *dosR*, *icl*, *acr* (*hspx*), and *lsr2*, which were demonstrated to be stimulated by hypoxia (Boon and Dick, 2002; Florczyk et al., 2003; Park et al., 2003; Starck et al., 2004; Fu and Fu-Liu, 2007), are expressed at low levels and/or are absent in the log-phase of bacterial growth. These genes were shown to be associated with dormancy of *Mycobacterium* (Boon and Dick, 2002; Park et al., 2003). Among the aforementioned genes, the dormancy survival regulon, regulated by the response regulator DosR, appears to be essential for hypoxic survival in a vast number of mycobacterial species, including *M. tuberculosis* (Park et al., 2003; Domenech et al., 2017), *Mycobacterium bovis BCG* (Alonso et al., 2011), and *Mycobacterium smegmatis* (*M. smegmatis*) (Homolka et al., 2009). DosR was shown to initiate transcription of an array of genes known as the *dosR* regulon, which comprises 48 genes containing the DosR-binding site and an ACP family sequence motif (Florczyk et al., 2003). The expression of these genes allows long-term survival of *M. tuberculosis* under anaerobic conditions in the host (Errey and Blanchard, 2005; Homolka et al., 2009; Alonso et al., 2011).

*Rv2629* is a member of the dosR dormancy regulon (Park et al., 2003) due to the presence of an ACP motif predicted by the Gibbs Recursive Sampler and SCAN (Florczyk et al., 2003). The *Rv2629* gene is conserved with regard to its expression in several environmental mycobacteria and related strains of *M. tuberculosis* (Wang et al., 2007). *Rv2629* has been reported to be induced by hypoxia and nitric oxide, while it is upregulated under dormancy conditions (Boon and Dick, 2002; Park et al., 2003; Voskuil et al., 2003; Starck et al., 2004), suggesting an association with the dormant state of *M. tuberculosis* (Starck et al., 2004). *Rv2629* was further identified as a drug-resistant protein by 2D-gel electrophoresis and MS analysis (Wang et al., 2007). A previous study determined the *Rv2629* sequence variations in 58 MDR strains and in 55 strains from a reference collection that represented the major *M. tuberculosis* complex pathogens namely, *M. tuberculosis* Beijing, *M. africanum* West African 1, *M. tuberculosis* (H37Rv; ATCC 27294), *M. bovis* (ATCC 19210), and *M. africanum* (ATCC 25420) (Homolka et al., 2009). Among these 58 MDR strains, 36 (62%) had the 191A/C mutation (codon 64, Asp to Ala) in *Rv2629* that supported an association with rifampicin resistance (Chakravorty et al., 2008; Homolka et al., 2009). The 191C allele was present in the Beijing strains when the MDR strains were stratified according to different phylogenetic lineages that indicated a classical Beijing IS*6110* restriction fragment length polymorphism pattern (Chakravorty et al., 2008; Homolka et al., 2009). The IS6*110*-restriction fragment length polymorphism (IS*6110*-RFLP) has been applied for the identification of the Beijing strains based on their hybridization patterns (Alonso et al., 2011). The Beijing strains evade the innate immune response and exhibit high capacity to cause and spread TB in humans compared with other *M. tuberculosis* lineages. Nevertheless, the exact mechanism of this action is yet to be determined (Alonso et al., 2011). Although the biological function of *Rv2629* remains unclear, it is speculated that its association with drug resistance involves maintenance of the persistent state of *M. tuberculosis*.

The present study aimed to investigate the function of the Rv2629 protein, notably the relationship between Rv2629 over-expression and bacterial physiological changes.

## Materials and Methods

### Strains and Growth Media

*Mycobacterium smegmatis* (MC2 155) and *M. tuberculosis* H37Ra were grown in Middlebrook 7H9 broth or Middlebrook 7H10 agar (DIFCO, United States) supplemented with 10% ADC (BD, United States) and kept in a biosafety P1 laboratory. The media for recombinant *M. smegmatis* and *M. tuberculosis* H37Ra that over-expressed *Rv2629* were supplemented with 50 μg/mL kanamycin, while for recombinant *M. smegmatis* that expressed low levels of *MSMEG_1130*, the medium was supplemented with 50 μg/mL kanamycin and 0.1% acetamide (w/v). Transformed *Escherichia coli* DH5α was cultured in Luria-Bertani broth and/or agar with the appropriate antibiotic (50 μg/mL kanamycin).

Strains isolated from clinical drug-resistant patients (strains 1570, 934, 945, 1573, 1176, 522, 1219, and 1221, all of the Beijing type) were obtained and cultured in a P2 laboratory at the Shanghai Pulmonary Hospital. All clinical strains included were rifampicin-resistant. Strain 934 was rifampicin (RIF) resistant with a minimum inhibitory concentrations (MIC) of 32 μg/mL. Strains 1570, 945, 1573, 1176, 1219, 522, and 1221 were MDR. All strains were identified and drug-susceptibility was evaluated as previously described (Ramaswamy et al., 2003; Wang et al., 2007). Clinical isolates and M. tuberculosis H37Rv were cultured under the same conditions as *M. tuberculosis* H37Ra (Kwon et al., 2017).

### Cell Culture

THP-1 cells were grown in house and were cultured in RPMI-1640 (Gibco, Foster City, CA, United States) supplemented with 10% (w/v) fetal bovine serum (FBS; Gibco) and 1% penicillin–streptomycin (Gibco). THP-1 cells were maintained at 37°C in a humidified atmosphere with 5% CO_2_.

### Cloning and Transformation

Recombinant plasmids encoding wild type *Rv2629* (*Rv2629^W^*) and the 191C mutant (*Rv2629^M^*) were constructed. The full length Rv2629 genes were amplified from genomic DNA extracted from H37Rv (for *Rv2629^W^*) and from clinical strains (for *Rv2629^M^*genotype). Recombinant plasmids encoding the antisense strand of *MSMEG_1130*, an ortholog of *Rv2629* in *M. smegmatis*, was constructed. The amplification was conducted by PCR using the appropriate primers (listed in Supplementary Table S1). The PCR products were digested with the corresponding enzymes (NEB, United States) (Supplementary Table S1) for 3 h at 37°C. Plasmid pMV261 (for over-expression) and/or pACT (an acetamide-inducible expression vector, for reduced expression) (Invitrogen, Waltham, MA, United States) were digested with the same restriction enzymes. The two fragments were ligated, transformed into *E. coli* DH5α and plated on LB agar containing kanamycin (50 μg/mL). Following overnight incubation at 37°C, single colonies were randomly selected and grown in LB broth. The plasmids were isolated from *E. coli* DH5α culture using an Axygen Miniprep Kit (Axygen, Union City, CA, United States) and confirmed by DNA sequencing.

The recombinant pMV261 and/or pACT plasmids were transformed into the *M. smegmatis* strain MC2 155 (*MS*) and/or *M. tuberculosis* H37Ra, respectively by electroporation (Reed et al., 2007). The study of *M. tuberculosis* genes in recombinant *M. smegmatis* is a recognized approach (Falcone et al., 1995; Daugelat et al., 2003; Andreu et al., 2004; Zimhony et al., 2004; Altaf et al., 2010; Sweeney et al., 2011; Bae et al., 2017; Sun et al., 2017; Yu et al., 2017; Wang et al., 2017). Recombinant *M. smegmatis* over-expressing *Rv2629* was identified as *MSW* (*Rv2629* wild type) and *MSM* (*Rv2629* mutant). Recombinant *M. tuberculosis* H37Ra over-expressing *Rv2629* was identified as *RaW* (*Rv2629* wild type) and *RaM* (*Rv2629* mutant). Recombinant *M. smegmatis* expressing low levels of *MSMEG_1130* was identified as MSL. Recombinant strains with the plasmid pMV261and/or pACT were constructed (*MSP, RaP*, or MS pACT) and used as controls. The types of strains with the corresponding genes and plasmids are presented in Supplementary Table S2.

### RNA Extraction and Real Time PCR (RT-qPCR)

Total RNA was isolated from *M. smegmatis* and *M. tuberculosis* using an RNA extraction kit (Omega, United States), according to the manufacturer’s instructions. Contaminating DNA was digested with RNase-free DNase I (Omega, United States) for 15 min at 37°C. Prior to further analysis the quality check of the RNA was determined by the estimation of the A260/A280 ratio. For RT-qPCR, cDNA was synthesized according to the manufacturer’s instructions (ReverTraAce qPCR RT kit, Toyobo, Japan).

qPCR analysis was conducted using *Rv2629*-specific primers (see Supplementary Table S1) with SYBR Green Real-Time PCR Master Mix (QPK-201) (Toyobo, Japan). 16S rRNA and Rv2703 (SigA) were used as reference sequences. The reaction conditions were: 94°C for 10 min, followed by 40 cycles of 94°C for 30 s, 56 to 65°C for 45 s, and 72°C for 30 s. A standard curve of copy number (range: 10^1^–10^8^) was constructed. Data were collected using a BIO-RAD iCycler (BIO-RAD, United States). RT-negative (without reverse transcriptase) reactions were used to account for residual DNA, and the copy numbers were normalized to those of 16S rRNA. The normalized values were used to determine the relative fold change in expression. The estimation of the relative expression of each gene was carried out using the ddCT method. The experiments were carried out in triplicate, and the results are expressed as mean ± SD.

### Western Blot Analysis

Whole cells were harvested from 20 mL cultures of *M. smegmatis* strains and *M. tuberculosis* isolates. The bacterial pellets were washed twice with 0.02 M PBS, resuspended, and sonicated in 200 μL of 0.02M PBS (supplemented with 1 mM PMSF and 10 mM EDTA) and centrifuged. The supernatant was treated with lysis buffer (8 M urea, 2 M thiourea, 140 mM DTT, 0.5% Biolyte pH 4–7, and 4% CHAPs). The protein concentration was estimated using the Bradford assay. Equal amounts of protein samples were separated by sodium dodecyl sulfate-polyacrylamide gel electrophoresis (SDS-PAGE, 12% gel), transferred to a PVDF membrane and detected with anti-Rv2629 rabbit antiserum (in house preparation) and/or the FtsZ monoclonal antibody (4°C, overnight) followed by a goat anti-rabbit IgG antibody conjugated with alkaline phosphatase (Sigma, United States) (37°C, 1 h). The blots were visualized with BCIP/NBT solution (room temperature, 20 min). The reaction was terminated by addition of deionized water. FtsZ was used as a loading control.

### Minimum Inhibitory Concentration Assay

Minimum inhibitory concentrations assay of recombinant *M. smegmatis* was carried out to detect the relationship between over-expressing Rv2629 proteins and the susceptibility to anti-TB drugs (streptomycin, isoniazid, rifampicin, ethambutol, ofloxacin, levofloxacin, moxifloxacin, amikacin, kanamycin, and capreomycin). Antibiotic dilutions and 96-well plate preparations (Falcon 3072; Becton Dickinson, Lincoln Park, NJ, United States) were carried out as described by Caviedes et al. (2002). Briefly, Rv2629 overexpressing strains were grown in 7H9/ADC at 37°C until an OD_600_ value of 0.5 was obtained. The bacterial suspensions were diluted 1000 times in 7H9 broth and the final diluted broth was dispensed into each well of a 96-well cell culture plate (100 μL/well). The drug storage solutions were diluted to prepare two-fold serial diluted solutions in 7H9 broth and subsequently added to the wells containing the bacterial suspensions. The plate was incubated for 48 h at 37°C. The wells containing bacterial pellets were denoted as the positive wells, whereas the wells containing clear broth were denoted as the negative wells. The minimum drug concentrations of the negative wells were the MICs of the drugs. All the experiments were carried out in triplicate.

### Microarray Analysis

Total RNA from each sample was quantified using the NanoDrop 1000 (Thermo Fisher, United States). RNA integrity was assessed using standard denaturing agarose gel electrophoresis. For microarray analysis, the Agilent Array platform was used according to the manufacturer’s standard protocols for sample preparation and microarray hybridization. Briefly, total RNA (1 μg) was amplified and transcribed into fluorescent cRNA, following the manufacturer’s Quick Amp Labeling protocol (Version 5.7, Agilent Technologies). The labeled samples were hybridized toward the *M. tuberculosis* H37Ra custom Oligo Array (8x15K, Agilent array) and were scanned using the Agilent Scanner G2505B (Agilent Technologies, United States). The Agilent Feature Extraction Software (version 10.5.1.1) (Agilent Technologies, United States) was used to analyze acquired array images. The median normalization and subsequent data processing was carried out using the GeneSpring GX v11.0 software package (Agilent Technologies, United States). Following Median normalization of the raw data, genes of at least one out of nine samples that exhibited flags in Present and/or Marginal (“All Targets Value”) were selected for further data analysis. Differentially expressed genes were identified using Volcano Plot filtering. Pathway and GO analyses were conducted in order to reveal the biological functions of this subset of differentially expressed genes. Finally, hierarchical clustering was carried out in order to show distinguishable gene expression profiling among samples.

### Transmission Electron Microscopy

For ultrathin cryo sections, log-phase *M. Smegmatis* cultures were harvested (5 mL). The cells were washed twice with 0.1 M phosphate buffer (PB) and fixed with 2.5% glutaraldehyde in 0.1 M PB (2 h). The log phase was estimated as determined previously (Belanger and Hatfull, 1999). Notably, a culture of *M. smegmatis* mc^2^155 was grown at 37°C, until an OD_600_ reading of approximately 0.8 was obtained. The cells were rinsed three times (15 min per wash) with 0.1 M PBS. After fixation in 1% osmium tetroxide (3 h), the cells were rinsed as previously described prior to dehydration with solutions containing increasing concentrations of ethanol and/or acetone at 4°C. The samples were subjected to gradual infiltration with Epon resin (3 days). Subsequently, the samples were solidified by incubation at increasing temperature for a total period of 2 days. Thin sections (70–80 nm) were prepared and stained with 3% uranyl acetate and lead citrate prior to examination under a Philips CM-120 transmission electron microscope (TEM) (Philips, United States).

### Growth Curve

To determine whether *Rv2629* expression levels among strains would lead to observable phenotypic growth and survival differences, recombinant *M. tuberculosis* H37Ra and *M. smegmatis* strains were subcultured thrice in Middlebrook 7H9 broth supplemented with 10% ADC and 0.05% Tween-80 (OD_600_ = 0.4). The *M. smegmatis* cultures were diluted when an OD_600_ value of 0.05 was obtained. Aliquots of 50 mL were dispensed in conical flasks and grown under continuous shaking at 220 rpm and aerobic conditions. The growth and survival were assayed by optical density readings (OD_600_) every 3 h following inoculation. The experiments were performed in triplicate.

The growth curve of recombinant *M. tuberculosis* H37Ra was detected using the BACTEC MGIT 960 Mycobacterial Detection System (Becton, Dickinson and Company, United States), as determined by the manufacturer’s instructions.

### Infection of THP-1 Cells with *M. tuberculosis* Strains and *M. smegmatis* Strains

THP-1 cells (5 × 10^5^) were stimulated with 5 ng/mL PMA (Sigma Chemical Co, St. Louis, MO, United States) for 24 h prior to infection. The infection was conducted using H37Rv, clinical *M. tuberculosis* strain 934, and recombinant *M. smegmatis* in the log-phase at a multiplicity of infection (MOI) value of 1:10 (37°C, 5% CO_2_, 4 h). Extracellular bacilli were removed by washing cells three times with PBS and the cells were cultured for 1 h in complete medium in the presence of 100 μg/mL gentamycin. The medium was replaced every 12 h during incubation, and the cells were lysed using 1% Triton X-100 solution at specific time points (0, 24, 48, 72, and 96 h). The precipitates were collected by centrifugation (2000 *g*, 20 min) for total RNA extraction, as described above. The number of CFU of intracellular mycobacteria and the viable bacteria present in the supernatant were determined on Middlebrook 7H10 agar with 10 % ADC.

### Mouse Infection

The culture of MSP and MSW was conducted in 5 mL of Middlebrook7H9 medium supplemented with 10% ADC and 500 mg sterile 2-mm glass beads at 37°C under continuous shaking. The cultures isolated during the log phase were centrifuged at 500 *g* in order to remove clumps, and the cells were washed twice with sterile saline. The cell suspension was adjusted to an OD_600_ of 0.8, corresponding to a concentration of 5 × 10^7^ CFU/mL. Female Balb/c mice (6–8-week-old) were infected intravenously with 0.1 mL of the bacterial suspension. The size of inoculum was confirmed by CFU counts.

The ability of survival and dissemination of each strain *in vivo* was assessed in six mice per group that were sacrificed by cervical dislocation on 0, 3, 6, 9, 12, 15, 20, and 35 days after infection. Lung, spleen, and kidney tissue specimens were weighed and homogenized in saline. The specimens were cultured in Middlebrook 7H10 medium (Becton Dickinson, United States) supplemented with 10% OADC following appropriate dilutions. CFUs were enumerated following 3–4 days of incubation at 37°C. All of the animal procedures were approved by the animal care committee of the Fudan University.

### 2D Gel Electrophoresis and MS Analysis

Whole cell extracts were prepared from 100 mL of cultures of each *M. smegmatis* strain in the log-phase. Bacterial pellets were washed twice and centrifuged (15°C, 8000 rpm, 15 min) in 0.02 M PBS (pH 7.4). The cells were sonicated in 500 μL of 0.02 PBS (0.02 M supplemented with 1 mM PMSF and 10 mM EDTA) and centrifuged at 12,000 rpm for 20 min at 4°C. The cells were treated with lysis buffer (8 M urea, 2 M thiourea, 140 mM DTT, 0.5% Biolyte pH 4-7, and 4% CHAPs) for 1 h and centrifuged (4°C, 12,000 rpm, 15 min). The supernatant was harvested and the protein concentration was estimated using the Bradford assay. Total protein (80 μg) were loaded on linear pH 4-7 IPG strips (Bio-Rad, United States) that were allowed to rehydrate at 50 V for 13 h. Isoelectric focusing was carried out using a Protean IEF (Bio-Rad, United States). The proteins were separated using 2-dimensional SDS-PAGE (12% gels) and stained with silver nitrate, as reported previously (Scheler et al., 1998; Gharahdaghi et al., 1999). The gels were scanned using Molecular Image FX (Bio-Rad, United States) and analyzed with the PD Quest 6.0 software (Bio-Rad, United States). The proteins of interest were excised from the silver-stained 2D gels and identified by MALDI-TOF-MS (Fu and Fu-Liu, 2007).

### Statistical Analysis

Statistical analysis was conducted using SPSS 11.5 (SPSS Inc., Chicago, IL, United States). Statistical differences of the continuous variables were analyzed by one-way analysis of variance (ANOVA) and the Student–Newman–Keuls test and/or Student *t*-test. The statistical level of significance was set at *P < 0.05*.

## Results

### Expression of Rv2629 in Different Clinical Strains

**FIGURE 1:**
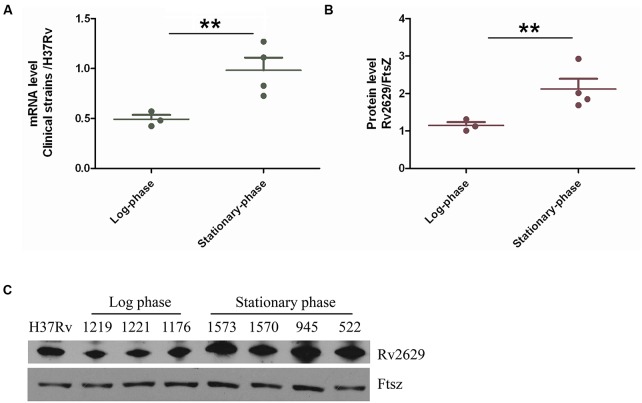
The expression of *Rv2629* was upregulated and down-regulated in clinical multidrug resistant (MDR) isolates in the stationary and log phases, respectively. **(A)** Total RNA was extracted from different drug-resistant clinical *M. tuberculosis* isolates in the log and stationary phases and was used RT-qPCR analysis. 16s RNA was used as the reference transcript. **(B)** Western blot analysis of *Rv2629* in the clinical MDR isolates at the log and stationary phases. **(C)** FtsZ was used as the loading control. The results are presented as the ratio of mRNA and/or protein level of *Rv2629* in clinical isolates compared with the corresponding ratio in *H37Rv*. The data are indicated as mean ± SEM. ^∗∗^*P* < 0.01.

Previous studies have demonstrated an increase in the expression of *Rv2629* at the transcriptional and/or translational levels induced by hypoxia and nitric oxide in clinical isolates of *M. tuberculosis* (Voskuil et al., 2003; Kassa et al., 2012; Zhang et al., 2014). Thus, we investigated the expression of the Rv2629 protein and mRNA levels among clinical isolates of *M. tuberculosis*. Quantitative RT-PCR was conducted using cDNA synthesized from total RNA isolated from bacilli from seven clinical strains (1573, 1570, 945, 1176, 1219, 522, and 1221) and the *M. tuberculosis* H37Rv control strain in the log and/or stationary-phases respectively (**Figure 1A**). The stationary phase was observed notably following 10 days of growth and the bacterial cultures were harvested at 3 h intervals for further analysis. It was observed that the average transcriptional level of Rv2629 in clinical isolates during the logarithmic phase was approximately two-fold lower than that noted in *M. tuberculosis* H37Rv, while during the stationary phase, this level was higher in the clinical isolates than in the control strain. The expression of Rv2629 at the protein level was further measured by Western blot analysis (**Figures 1B,C**). The cytoplasmic fraction of the cell extracts obtained from the clinical isolates indicated a two-fold increase in the stationary phase compared to that noted in *M. tuberculosis* H37Rv. The over-expression of Rv2629 at both the protein and the mRNA levels in clinical *M. tuberculosis* isolates was related to the stationary phases of the strains. Rv2629 exhibited reduced expression during the log-phase, when bacilli were actively duplicating.

### Expression of Rv2629 in Clinical *M. tuberculosis* Strain 934 in THP-1 Cells

**FIGURE 2:**
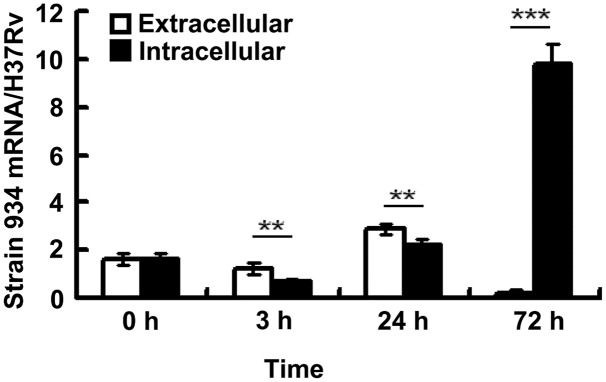
*Rv2629* mRNA was upregulated in RIF-resistant clinical *M. tuberculosis* strains following infection in THP-1 cells. A total of 5 × 10^5^ THP-1 cells were infected with H37Rv or, a clinical rifampicin-resistant isolate (strain 934) in the log-phase at multiplicity of infection (MOI) = 10, following stimulation with 5 ng/mL of PMA for 24 h. The mRNA levels of *Rv2629* were measured at 0, 3, 24, and 72 h (intracellular group). Strain 934 was cultured in the absence of THP-1 cells and served as control (extracellular group). The results are presented as the ratio of mRNA levels of *Rv2629* in strains 934 and H37Rv. The expression of intracellular *Rv2629* mRNA was 45-fold higher than that noted in the extracellular 72 h post-infection. The data are presented as mean ± SEM. ^∗∗^*P* < 0.01, ^∗∗∗^*P* < 0.001. The experiments were performed in triplicate.

In order to investigate the association of Rv2629 with dormancy, the THP-1 human leukemic monocyte cell line was infected with H37Rv and/or a clinical RIF-resistant isolate 934 in the log-phase at MOI = 10, following stimulation with 5 ng/mL PMA for 24 h. The mRNA levels of *Rv2629* expressed in bacteria were measured at 0, 3, 24, and 72 h. Strain 934 was cultured in the absence of THP-1 cells and served as control. Rv2629 gene intracellular expression was further determined during several time periods (**Figure 2**). The results indicated that the ratios of the Rv2629 mRNA levels of strain 934 to H37Rv at 3 and 24 h were higher in the extracellular compared with the intracellular compartment (*P* < 0.01), whereas the opposite was noted for the 72 h period (*P* < 0.001) (**Figure 2**). Moreover, a high upregulation (45-fold) of intracellular *Rv2629* expression in the clinical strain was observed at 72 h post-infection compared with the extracellular control (**Figure 2**), supporting the hypothesis that intracellular bacilli can remain in a dormant phenotype up to day 3 by over-expressing Rv2629.

### Confirmation of Overexpression and Reduced-Expression of Rv2629 in *Mycobacteria*

**FIGURE 3:**
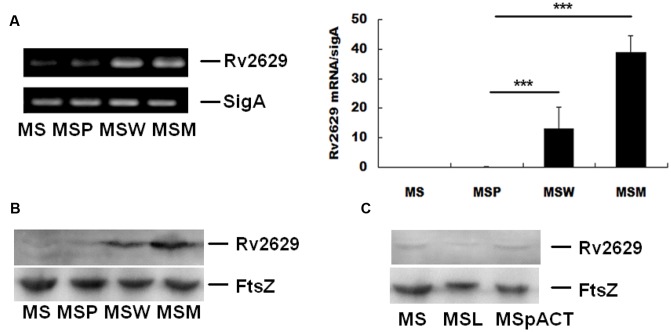
Overexpression and reduced expression of wild type *Rv2629* and/or mutant *Rv2629* in *M. smegmatis*. **(A)** qRT-PCR using total RNA extracted from four *M. smegmatis* strains, namely, *MSW* (wild type *Rv2629*), *MSM* (191A/C mutant), MS, and MSP (control strains). **(B)** Western blot analysis of over-expression of *Rv2629* in the four *M. smegmatis* strains. **(C)** Western blot analysis of reduced expression of *Rv2629* in MSL and MSpACT. The data are presented as mean ± SEM. ^∗∗∗^*P* < 0.001. The experiments were performed in triplicate.

*M. smegmatis* over-expressing *Rv2629^W^* (*MSW*) and *Rv2629^M^* (*MSM*) were produced in order to simulate *Rv2629* expression levels in clinical *M. tuberculosis* strains and investigate the function of the *Rv2629* protein. Western blot and RT-qPCR analysis of four *M. smegmatis* strains during the log-phase confirmed the increased expression of *Rv2629* in the *MSW* and *MSM* compared with the control *MS* and *MSP* strains (**Figures 3A,B**).

The ortholog of *Rv2629* in *H37Ra* and *M. smegmatis* strains are *MRA_2657* and *MSMEG_1130*, respectively. The DNA and amino acid sequences of *MRA_2657* are completely identical with *Rv2629*, while *MSMEG_1130* and *Rv2629* are highly homologous, in which the overlap value between *MSMEG_1130* and *Rv2629* was 366 based on the data of KEGG database. *M. smegmatis* MSL corresponded to a bacterial strain expressing low levels of *MSMEG_1130*. The strain was constructed to investigate the function of *Rv2629*, whereas the plasmid pACT, an acetamide-inducible expression vector, served as control. For generating the MSL strain, the plasmid pACT was subjected to recombination with the DNA fragment, whose mRNA production could complement with the *MSMEG_1130* mRNA sequence reversely and thus could inhibit the expression of *MSMEG_1130* gene. Western blot analysis in the two *M. smegmatis* strains during log-phase confirmed low expression of *MSMEG_1130* in MSL compared with control MSpACT (**Figure 3C**). The use of *M. smegmatis* as a parental strain for the overexpression of *Rv2629* has been reported in a previous study (Wang et al., 2007).

### Minimum Inhibitory Concentration Assay of Recombinant *M. smegmatis*

In order to investigate the association of *Rv2629* with drug-resistance, 10 anti-TB drugs were selected including four first-line drugs (streptomycin, isoniazid, rifampicin, and ethambutol) and six second-line drugs (ofloxacin, levofloxacin, moxifloxacin, amikacin, kanamycin, and capreomycin). The MIC of recombinant *M. Smegmatis* was determined. The results are shown in **Table 1**. The Rv2629 over-expressing strains enhanced drug-resistance of recombinant *M. smegmatis* to ethambutol, moxifloxacin, and levofloxacin at least two-fold compared with the MSP strain.

**Table 1:** Minimal inhibition concentrations (MICs) of recombinant *M. smegmatis* against first- and second-line drug treatment.

	MSW	MSM	MSP
Streptomycin	0.5	0.5	0.5
Isoniazid	4	4	4
Rifampicin	4	2–4	2–4
Ethambutol	0.5	0.5	≤0.25
Ofloxacin	0.25	0.25	0.25
Levofloxacin	0.25	0.25	≤0.125
Moxifloxacin	0.125	0.125	≤0.06
Amikacin	1	1	1
Kanamycin	>32	>32	>32
Capreomycin	4	4	8


### Microarray Analysis of *M. tuberculosis* H37Ra Overexpressing Rv2629

**FIGURE 4:**
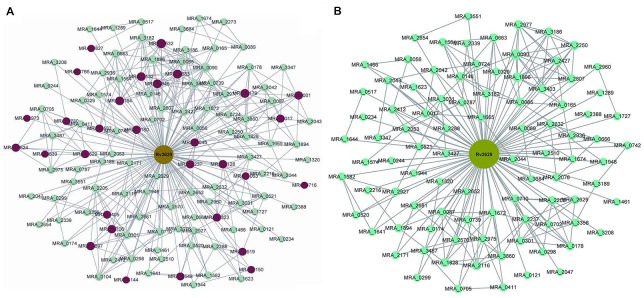
Interaction network of genes that correlated with Rv2629 and/or 191A/C mutated *Rv2629* in *RaW* and/or *RaM* strains. **(A)** Module A indicates the interaction of the genes associated with the wild type Rv2629 in *RaW*. **(B)** Module B indicates the interaction of genes associated with the mutated Rv2629 (191A/C mutation) *RaM* strain. Taking into account the correlation coefficient between each particular gene and these two modules, two genes are connected if the correlation is greater than a selected threshold (0.9). Genes are shown in different sizes. A larger size (122 genes) indicates more connections with other modules; a smaller size (92 genes) indicates fewer connections. Different types of genes are in diverse colors. Rv2629 is the target gene of the present study. Green nodes are included in two conditions. Red nodes represent genes only in *RaW* and not in *RaM* strains.

The present study aimed to investigate the differences in gene expression caused by *Rv2629* overexpression in *M. tuberculosis* strains and the potential interplay in the biological function of the *Rv2629* gene. Microarray analysis was conducted on recombinant *M. tuberculosis* H37Ra overexpressing *Rv2629* in the log phase. The results indicated that recombinant *M. tuberculosis H37Ra* overexpressing Rv2629 (*RaW*) and *H37Ra* overexpressing 191 A/C mutated Rv2629 (*RaM*) remained in the log phase of growth, respectively. In addition, 122 genes correlated with wild type *Rv2629* (Supplementary Table S3), whereas 92 genes correlated with 191A/C mutated *Rv2629*. The genes from the two strains were shown in different sizes (**Figure 4**). As shown in **Figure 4**, two genes are connected if the correlation between them is greater than a selected threshold (0.9). The correlation is based on a correlation coefficient between each particular gene and the two modules. A total of 30 genes correlated with wild type *Rv2629* and to a lesser extent with 191 A/C mutated *Rv2629* (**Table 2**). The correlation coefficient was lower than the selected threshold in *RaM*. The remaining 92 genes correlated with *Rv2629* in the two bacterial strains.

**Table 2:** Thirty genes solely correlated with wild type *Rv2629.*

Probe Name	Protein ID	Annotation	RaW vs. Control	RaM vs. Control	RaW vs. RaM
MRA_3766	YP_001285117.1	Hypothetical protein	Up	Up	Up
MRA_3537	YP_001284884.1	MCE-family protein Mce4C	Up	Down	Up
MRA_3532	YP_001284879.1	Mce associated protein	Up	Up	Up
MRA_3405	YP_001284751.1	Hypothetical protein	Up	Up	Up
MRA_3184	YP_001284529.1	NADH dehydrogenase subunit G	Up	Up	Up
MRA_3180	YP_001284525.1	NADH dehydrogenase subunit C	Up	Up	Up
MRA_3179	YP_001284524.1	NADH dehydrogenase subunit B	Up	Up	Up
MRA_3144	YP_001284489.1	Molybdenum cofactor biosynthesis protein D1	Up	down	up
MRA_3063	YP_001284407.1	Glycosyltransferase	Up	Up	Up
MRA_2973	YP_001284316.1	Putative polyketide synthase Pks1	Up	Up	Up
MRA_2846	YP_001284187.1	Hypothetical protein	Up	Up	Down
MRA_2273	YP_001283597.1	Putative secreted protein	Up	Up	Up
MRA_2128	YP_001283451.1	Putative integral membrane protein	Up	Down	Up
MRA_2045	YP_001283367.1	Hypothetical protein	Up	Down	Up
MRA_1897	YP_001283215.1	Secreted antigen 85-B FbpB	Up	Down	Up
MRA_1706	YP_001283017.1	Hypothetical protein	Up	Up	Up
MRA_1653	YP_001282961.1	50S ribosomal protein L35	Up	Up	Up
MRA_1624	YP_001282932.1	Prolipoprotein diacylglyceryl transferase	Up	Up	Up
MRA_1622	YP_001282930.1	Tryptophan synthase subunit beta	Up	Up	Up
MRA_1323	YP_001282626.1	UDP-*N*-acetylglucosamine 1-carboxyvinyltransferase	Up	Up	Up
MRA_1132	YP_001282432.1	Putative peroxidase BpoB	Up	Down	Up
MRA_1001	YP_001282298.1	Molybdopterin biosynthesis protein MoeA1	Up	Up	Up
MRA_0827	YP_001282118.1	Hypothetical protein	Up	Up	Up
MRA_0730	YP_001282020.1	50S ribosomal protein L30	Up	Up	Up
MRA_0716	YP_001282006.1	50S ribosomal protein L16	Up	Up	Up
MRA_0639	YP_001281925.1	Exodeoxyribonuclease V subunit beta	Up	Up	Up
MRA_0619	YP_001281905.1	Hypothetical protein	Up	Up	Up
MRA_0548	YP_001281833.1	Putative integral membrane protein	Up	Up	Up
MRA_0150	YP_001281430.1	Chloride channel	Up	Up	Up
MRA_0104	YP_001281385.1	Hypothetical protein	Up	Up	Up


The GO and pathway analysis indicated that 122 genes correlated with wild type *Rv2629*; they were involved in ribosome construction, toluene degradation, arginine and proline metabolism, oxidative phosphorylation, and homologous recombination. The genes were notably related to the ribosome construction and the Fisher *P*-value was 4.28 × 10^-10^ (Supplementary Table S4). The genes that solely correlated with wild type *Rv2629* were notably related to ribosome production, oxidative phosphorylation, and virulence (**Table 2**). The results indicated that the 191A/C mutation in *Rv2629* could influence the function of wild type *Rv2629* partially. The mutation may lead to the decreased effect of Rv2629 on ribosome production, oxidative phosphorylation, and virulence.

### Reduced Ribosome Content in *M. smegmatis* Overexpressing Rv2629

**FIGURE 5:**
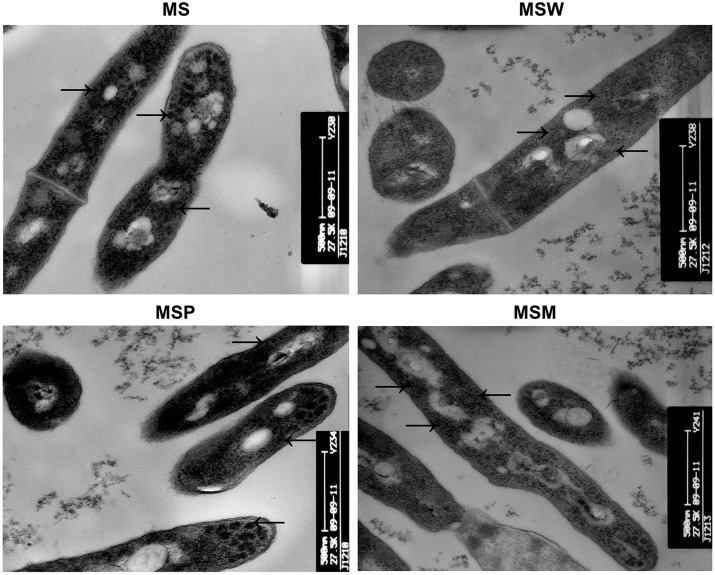
Transmission electron microscope analysis of ribosomal content. The ribosomal content (arrows) of *MS, MSW, MSP, and MSM* strains. The data are representative of two independent experiments with three samples in each experiment. Five random fields were observed.

The effects of the *Rv2629* gene on the morphological features of MSW and MSM at the log-phase were investigated by TEM, as demonstrated by previous studies (Tumminia et al., 1991). No difference was noted in cell wall thickness and morphology, although recombinant *M. smegmatis* MSW revealed apparent differences in their cytoplasmic component compared to the MSM, MSP, and MS strains (**Figure 5**). The cytoplasm of MSW cells exhibited lower electron density and the cytoplasmic ribosomal content was reduced significantly compared to the MS and MSP strains, with the exception of the MSM strain (**Figure 5**).

### Growth Characteristic of Recombinant *M. smegmatis*

**FIGURE 6:**
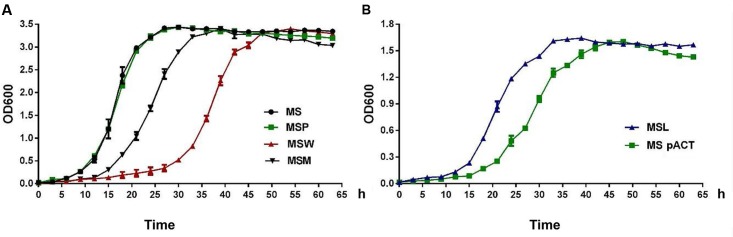
The expression of *Rv2629* modulates the growth of *M. smegmatis* under aerobic conditions. The growth curve of recombinant *M. smegmatis* strains that exhibited reduced expression of *Rv2629* and overexpression of the *Rv2629^W^* and/or the *Rv2629^M^* genes, respectively. The growth of the strains was measured by harvesting cultures at 3-h intervals and subsequent measurement of the absorbance values at 600 nm (OD_600_). The abbreviations were: *MS* (wild type), *MSP* (control strain transformed with the pMV261 plasmid), and *MSpACT* (control strain transformed with the pACT plasmid). The results indicated **(A)** comparatively slow growth of *MSW*. **(B)** Comparatively rapid growth of *MSL*. The data are representative of two independent experiments with two samples in each experiment which shows similar results.

The microarray results indicated that Rv2629 might be related to ribosome production. Consequently, the growth curves of the *MSW* and *MSM* strains were investigated by harvesting cultures at 3-h intervals and by measuring the OD_600_. *MS* and *MSP* served as controls. The results of the two independent experiments demonstrated comparatively slow growth of *MSW* (**Figure 6A**) and indicated that *Rv2629* overexpression inhibits bacterial growth and delays entry in the log phase. The growth of *MSM* was more rapid compared with *MSW* and slower than *MSP* and *MS*, which suggested that the 191A/C mutation of the *Rv2629* gene weakens the inhibitory function of Rv2629. A similar effect was noted in the *RaW* and *RaM* strains (**Supplementary Figure S1**).

MSL strains that expressed low levels of *MSMEG_1130* were investigated, as described above. *MSpACT* was used as the control. The results of two independent experiments indicated comparatively rapid growth of *MSL* (**Figure 6B**), while the low expression of *MSMEG_1130* promoted bacterial growth and entry to the log phase.

**FIGURE 7:**
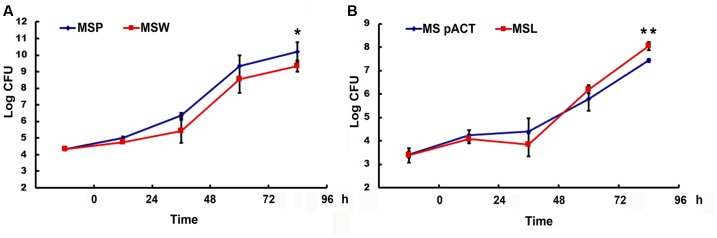
The expression of *Rv2629* influences the growth of *M. smegmatis* in human THP-1 cells. **(A)** *M. smegmatis* over-expression of *Rv2629* (*MSW*) resulted in the slow growth of THP-1 following infection (*P* < 0.01, 96 h). **(B)** *M. smegmatis* low-expressing *MSMEG_1130* (*MSL*) strain exhibited a rapid growth in THP-1 cells at 96 h post-infection, *P* < 0.01 (96 h). The data of each time point was analyzed by the Student’s *t*-test after homogeneity test of variance, *n* = 4. The experiments were performed in triplicate.

The enhanced and/or attenuated *in vitro* growth phenotypes of recombinant *M. smegmatis* prompted the examination of the correlation between *Rv2629* expression and dormant phenotype. THP-1 cells were infected with the bacteria. No major differences in the intracellular number of viable bacteria were observed at 24 h, suggesting that recombinant *M. smegmatis* strains that expressed high and low levels of *Rv2629* exhibited similar rates of growth to that of control strains within the first day after the infection (**Figure 7A**). Nevertheless, the bacterial growth of the over-expressing *Rv2629* strains was impaired at day 2 post-infection, whereas the viable bacilli number at the turn point of growth from lag to the log-phase was decreased. This result is consistent to that found in the *in vitro* experiments. Furthermore, over-expression of Rv2629 decreased the growth rate of the bacilli and further prevented them from achieving higher cell density in the stationary phase. This conclusion was further confirmed by comparison of the growth capability observed in THP1 cells of low-expressing the *MSMEG_1130 M. smegmatis* strains. The bacilli showed increased growth rate compared with the control samples from 48 to 96 h post-infection (**Figure 7B**). The growth of MSL in THP-1 cells was evaluated at MOI values of 1:5 and 1:20. The results were consistent with the results observed at MOI of 1:10 (**Supplementary Figure S2**).

### Growth and Pathogenicity of Rv2629 Overexpressing *M. smegmatis* Strains in Mice

**FIGURE 8:**
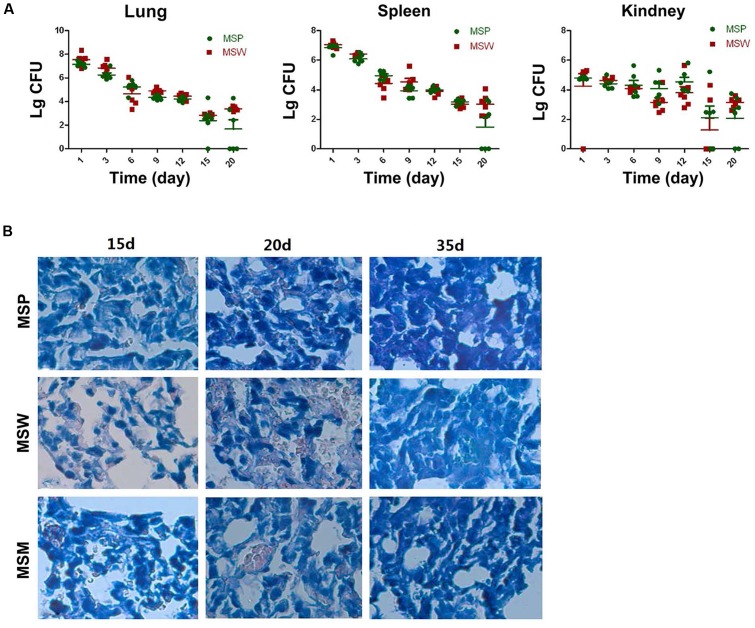
The growth of *M. smegmatis* strains in different organs of Balb/c mice. **(A)** Colony forming units (CFU) of *M. smegmatis* in lung, spleen, and kidney tissues of mice infected with *M. smegmatis* at different time points (0, 3, 6, 9, 12, 15, and 20 days post-infection). **(B)** Acid-fast staining of lung tissues of mice infected with different *M. smegmatis* strains (400×) at different time points (15, 20, and 35 days post-infection). The data are representative of two independent experiments, *n* = 6.

**FIGURE 9:**
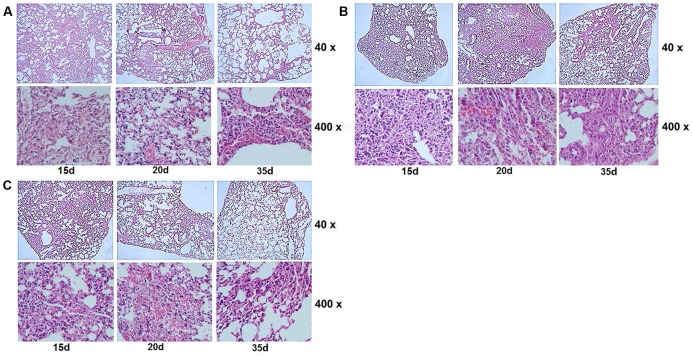
Pathological changes in the lung tissues of each group at different time points (15, 20, and 35 d) post-infection. Images **(A–C)** indicate the lung tissues of mice infected with MSP, MSW, and MSM, respectively. The structure of pulmonary alveolus of the mice infected by MSW was permeated with inflammatory exudates and infiltrated by inflammatory cells. The alveolar wall indicated congestion and incrassation, with apparent granuloma formation in the lungs at 15 and 20 days post-infection. The data are representative of two independent experiments, *n* = 6.

The attenuated reproductive capacity of *MSW* in macrophage infection prompted us to investigate whether MSW could further decrease the virulence and pathogenicity of host tissues. Balb/c mice were infected with MSP, MSW, and MSM in order to identify the different pathogenicity and survival ability among these strains *in vivo*. The CFU values of bacteria in the lung, spleen, and kidney tissues were counted. The results indicated that the MSW strain exhibited a longer survival time, notably in the lung tissues compared with MSP, while with regard to the MSW strain the detection of the bacterial growth was evident in the lungs of the mice following 20 days of infection (**Figure 8A**). This observation occurred despite the absence of CFU counts between MSP and MSW at each time point. The acid-fast staining of lung tissue indicated that overexpression of wild type *Rv2629* could promote the survival of *M. smegmatis* in granulomas of the lung. No significant differences among the three strains were noted at the 15th day after infection. The number of MSW strains that overexpressed the wild type Rv2629 increased significantly compared to the other two strains in the granuloma of the lung at the 20th day after infection. No visible MSP and/or MSM could be found in the diseased location at the 35th day post-infection, although a high number of MSW strains formed granulomas in the lung (**Figure 8B**). Additional pathological tests indicated that the pathogenicity of MSW increased and additional damage was caused in the lung tissues compared to the MSP and/or MSM strains. The pulmonary alveolus of mice infected with MSP was damaged slightly and it was the sole cause of bleeding in the bronchial lumen in the absence of granuloma formation during the 15th day following infection. Sporadic granuloma could be found in the small part of the lungs at the 20th day after infection, whereas the inflammatory exudate was absorbed and the main part of the lung recovered to the normal structure 35 days post-infection (**Figure 9A**). Apparent changes could be observed in the lung of MSW-infected mice. The normal structure of the major part of the pulmonary alveolus disappeared, which was permeated with inflammatory exudate and associated with the infiltration of a great number of inflammatory cells. The alveolar wall indicated congestion and incrassation, whereas apparent granuloma could be found in the lungs 15 days post-infection. The area and degree of damage increased further 20 days post-infection, and the normal structure of the complete part of the pulmonary alveolus disappeared. The structure of the majority of the pulmonary alveolus recovered, although a certain part of granuloma could be found in the lung (**Figure 9B**). The pathogenicity that was caused by the Rv2629 expressing strains decreased when a missense mutation (Asp64 to Ala64, A/C) was present at position 191 of the genetic sequence of *Rv2629*. The pulmonary alveolus of mice infected with MSM was damaged partly and sporadic granuloma could be found in certain parts of the lungs 15 days post-infection. The damaged structure of pulmonary alveolus recovered gradually and the majority of the lung tissue recovered to the normal structure at the 20th and the 35th day week post-infection, respectively, as noted for the MSP strain (**Figure 9C**). Consequently, the overexpression of the wild type *Rv2629* could increase the survival and pathogenicity of *M. smegmatis*, whereas the 191 A/C mutation in *Rv2629* could weaken the pathogenicity of the *Rv2629*-expressing strains.

### Identification of Protein Expression by MALDI- TOF-MS in *M. smegmatis* Overexpressing Rv2629

**FIGURE 10:**
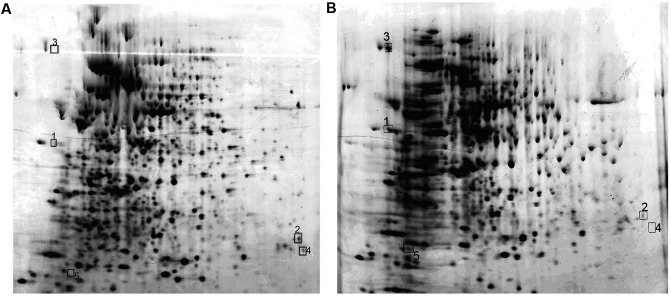
2-DE patterns of whole cell supernatant proteins from *MSP* and *MSW* strains that were conducted in the pH range of 4–7. The proteins from *MSP* **(A)** and *MSW* **(B)** were visualized by silver staining during the late log phase of bacterial growth. The spot numbers on the reverse image correspond to proteins identified by MALDI-TOF-MS, as listed in **Table 3**. The data are representative of two independent experiments.

Protein expression maps of *M. smegmatis* overexpressing *Rv2629* were obtained by 2-DE (**Figure 10**). A total of eight differential proteins were found by comparing the protein differential expressing map on 2-DE gels. A total of five proteins, namely, CHP, UK, TF, AA, and FFP, were identified by MALDI-TOF-MS (**Table 3**). Nevertheless, the expression pattern of these proteins differed among the strains. TF and FFP were present only on the *MSW* 2-DE gel, as opposed to the *MSP* gel. AA was not detected on the *MSW* 2-DE gel. The expression of UK decreased, while the expression of CHP increased in the MSW compared with the *MSP* strains.

**Table 3:** Proteins expressed differentially between *MSW* and *MSP.*

Spot	Gene	GI in NCBI*^a^*	Locus tag*^a^*	Peptide sequence matched	Protein	Expression
no.				by MS/MS	score	level*^b^*
1	Conserved hypothetical protein	gi| 118174177	MSMEG_2381	AGTLIAAVPLDPALSVAGAER	74	Increased
2	Uridylate kinase	gi| 118170082	MSMEG_2540	TNPDAELITAISHR	203	Decreased
3	Trigger factor	gi| 118173001	MSMEG_2540	GAVLEQVVNDALPSR	318	New
4	Amino-acid acetyltransferase	gi| 118172835	MSMEG_5612	AADDGLSFVVR	100	Deletion
5	Ferritin family protein	gi| 118170246	MSMEG_6422	DALALALDQER	134	New


*^a^GI in NCBI and locus tag according to the complete genome of *M. smegmatis* strain *MC2 155* (http://www.ncbi.nlm.nih.gov). ^b^Expression of identified proteins in *MSW* compared with *MSP*.*

## Discussion

In the present study, an expression analysis of the Rv2629 native protein was conducted in recombinant *M. smegmatis* and *M. tuberculosis* strains and in clinical isolates. In addition, the growth characteristics of recombinant *M. smegmatis*, expressing *Rv2629* wild type and mutant genes were evaluated *in vitro* and *in vivo*. It is suggested that *Rv2629* is correlated with the survival of the clinical drug-resistant *M. tuberculosis* strains via the repression of bacterial growth and the induction of bacterial persistence.

It has long been speculated that a latent and/or dormant state may play a role in drug tolerance of *M. tuberculosis* (Florczyk et al., 2003; Starck et al., 2004; Errey and Blanchard, 2005; Bartek et al., 2009; Peddireddy et al., 2017). The first-line antibiotics used to treat TB are all active against replicating bacteria (Hoffmann et al., 2010) but not against anaerobic dormant bacilli (Ramaswamy et al., 2003; Fu and Fu-Liu, 2007). The fact that the *DosR* regulon is required for long-term survival under anaerobic conditions has led to the notion that *DosR*-regulated genes may contribute to the higher level of *M. tuberculosis* drug tolerance during infection (Boon and Dick, 2002; Florczyk et al., 2003; Ramaswamy et al., 2003; Starck et al., 2004; Errey and Blanchard, 2005; Bartek et al., 2009; Alonso et al., 2011; Peddireddy et al., 2017). The present study indicated that *Rv2629* expression was decreased in clinical drug-resistant isolates during the log-phase compared with H37Rv, although the expression of the protein was increased during the stationary-phase. It can be speculated that upregulation of *Rv2629* in the stationary-phase is beneficial to the survival of drug-resistant strains *in vivo*, and therefore the protein may be indirectly associated with drug resistance. The drug tolerance observed in the present study may be due to alternative mechanisms, such as the survival of the bacilli in a non-replicating and/or low metabolic state environment.

The present study indicated that overexpression of *Rv2629* may inhibit the biosynthesis of *M. smegmatis* in the lag-phase, during which the synthesis of the enzymes and proteins required for metabolism and rapid reproduction occurs. TEM indicated that the ribosome content was decreased in *MSW* in the log phase compared with the *MSP* strain. Furthermore, 2D-gel electrophoresis indicated a decrease of UK and a deletion of AA proteins in *MSW* compared with the *MSP* strain. The enzymes are known to be involved in pyridine synthesis and arginine metabolism in *M. tuberculosis* (Errey and Blanchard, 2005; Rostirolla et al., 2011). UKs, also known as UMP kinases, are key enzymes in the synthesis of nucleoside triphosphates. The members of the UMP kinase family catalyze the conversion of UMP to UDP, an essential step in the pyrimidine metabolic pathway in a variety of bacteria (Yoshida et al., 2012). In many bacterial genomes, the gene tends to be located immediately downstream of elongation factor T and upstream of ribosome recycling factor. This enzyme has been shown to be essential in bacteria. The product of *pyrH* (*Rv2883*) gene, a member of the UMP kinase family, has been shown to be essential for *M. tuberculosis* growth by the rapid screening method (Lee et al., 2007; Rostirolla et al., 2011). Genetic studies have provided evidence that UMP kinases are essential for the growth of both Gram-negative (e.g., *E. coli*) and Gram-positive bacteria (e.g., *Streptococcus pneumoniae*) (Yamanaka et al., 1992; Fassy et al., 2004). Therefore, it can be speculated that *Rv2629* delays entry in the log-phase by inhibiting protein biosynthesis. Delayed growth under conditions of dormancy is likely to be beneficial for the survival of bacteria in the host, and therefore may contribute to the increased pathogenicity of the strain. Although Rv2629 has been characterized as a dormancy protein that is induced under anaerobic conditions of *M. tuberculosis*, the main focus of research has been toward the ability of this protein to confer resistance to bacterial strains (Florczyk et al., 2003; Wang et al., 2007). The current study investigates the novel hypothesis that the reduced growth and dormancy-type characteristics of *M. smegmatis* and *M. tuberculosis* are not entirely dependent on antibiotic resistance. The drug tolerance observed in the present study may be due to alternative mechanisms, such as the survival of the bacilli in a non-replicating and/or low metabolic state. Similarly, it has been reported that the phenotypic tolerance acquired in *M. tuberculosis* to isoniazid treatment is via the activation of mycobacterial DNA activating protein (MDP1) and the regulation of kat G transcription (Niki et al., 2012).

The aforementioned results supported the functional overlap of *Rv2629* between *M. smegmatis* and *M. tuberculosis*. The overlap between the two strains has been proposed by a previous study (Homolka et al., 2009). The *dosR* gene exhibited a severe survival defect in *M. smegmatis* under hypoxic conditions and this phenotype could be complemented by the *dosR* gene from *M. tuberculosis*, which supported the functional overlap between *DosR* from *M. smegmatis* and *M. tuberculosis* (Boon and Dick, 2002; Park et al., 2003; Homolka et al., 2009).

The pathogenicity of the *Rv2629* overexpressing *M. smegmatis* strain in the tissues of BalB/c mice was investigated and it was found that the overexpression of *Rv2629* increased the bacterial survival in the lung and spleen organs. The exact cause of this effect remains unknown. Microarray analysis indicated that the mRNA levels of several proteins, namely, MCE-family protein Mce4C (MRA_3537), secreted antigen 85-B FbpB (MRA_1897), and Mce associated protein (MRA_3532), were upregulated in the recombinant strain wild type *Rv2629* compared with the *Rv2629* mutant strain, which might be associated with the increased pathogenicity and dormancy resistance. *Mce4* encodes a cholesterol transporter that is associated with cholesterol metabolism and is essential for mycobacterial persistence. The disruption of *mce4* results in failure of *M. tuberculosis* to maintain chronic infection in mice, while retaining full virulence during the acute phase (Mohn et al., 2008; Pandey and Sassetti, 2008). In contrast to these findings, the MCE family protein and the Mce-associated protein are generally considered as virulence factors, and are associated with lipid transport and host cell invasion (Rodriguez et al., 2015; Perkowski et al., 2016). Ag85B may also contribute to the adherence, invasion, and dissemination of organisms in the host tissue, which can bind to Fn and play a critical role in mycobacterial adherence to the host cells (Kassa et al., 2012). Furthermore, Ag85B serves as an important colonization factor potentially contributing to mycobacterial virulence (Kuo et al., 2012). To date, no proteomic studies have been conducted with regard to the *Rv2629* mutant strain of *M. smegmatis* and/or *M. tuberculosis*. The 2D-gel electrophoresis indicated that the proteins TF and FFP were expressed in *MSW*, but not in *MSP* strains. TF is a FK506 binding protein (FKBP)-type peptidyl-prolyl cis-trans isomerase (PPIase) that is considered a highly conserved ribosome-associated chaperone found in a vast number of bacteria (Hoffmann et al., 2010). This protein has been reported to be essential for protein synthesis and survival under conditions of stress (Wu et al., 2011). The present study indicated that overexpression of *Rv2629* induced the expression of TF and FFP and might improve bacterial survival in the persistence state.

The success of the pathogenicity of *M. tuberculosis* is largely attributed to its ability to manipulate the host immune responses. The genome of *M. tuberculosis* encodes multiple immune-modulatory proteins that have been implicated in the pathogenicity of this strain. In agreement with the main concept of the current study, it has been shown that the immune-modulatory protein PE_PGRS41 boosted the survival of *M. smegmatis* within macrophages and was accompanied by enhanced cytotoxic cell death via inhibition of apoptosis and autophagy. This indicated that specific bacterial proteins can act as virulence factors that enhance the pathogenic properties of *M. smegmatis* (Deng et al., 2017). To the best of our knowledge the implication of the antigenic protein Rv2629 in the virulence of *M. smegmatis* has not been reported to date. The number of studies that have examined the contribution of this antigen in the immune response elicited against *M. tuberculosis* is very limited (eight in total). The majority of the studies on Rv2629 have focused on the contribution of this protein to the immune response elicited against *M. tuberculosis* as well as in the resistance developed against agents such as Rifampicin (Chakravorty et al., 2008; Louw et al., 2009; Niki et al., 2012). In addition, polymorphisms of *Rv2629* have been used as potential markers for the identification process of certain *M. tuberculosis* genotypes (Alonso et al., 2011; Zhang et al., 2014). Consequently, the current study offers novel evidence on the potential interplay of the Rv2629 protein regarding the survival of *M. tuberculosis*.

Based on KEGG database, the overlap value between MSMEG_1130 and Rv2629 was 366. Moreover, Blastn data showed that there are 54% identical nucleotide sequences and Blastp data showed that there are 59% similar or identical amino acid sequences between them. The nucleotide and amino acid sequences of two genes are similar, there was certain homology of nucleotide and amino acid sequences between the two genes. Therefore, MSMEG_1130 was the most homologous protein with Rv2629 in *M. smegmatis* strains, with more than 50% amino acids identical or similar with Rv2629. To further clarify the problem, other low-expression models such as the MRA_2657 low-expression H37Ra strain should be further investigated. Furthermore, there was no experimental data supporting the different function of MSMEG 1130 from Rv2629. On the contrary, MSMEG_1130 might have similar function with Rv2629 related with the growth of *M. smegmatis*, for our data show MSMEG_1130 low-expression strain exhibited a rapid growth compared with the control. MSMEG_1130 was also considered as a protein related with the growth of *M. smegmatis* in other study (Lauten et al., 2010).

## Conclusion

The present study strongly suggests that the overexpression of the wild type Rv2629 protein could delay the growth of the *M. smegmatis* by modifying the expression of specific proteins involved in bacterial metabolism. The overexpression of both WT and mutant *Rv2629* did not affect the susceptibility of *M. smegmatis* to antibiotics, although it increased its survival and pathogenicity *in vivo*. The findings propose a potential explanation regarding the association of the protein Rv2629 with the survival of the clinical drug resistant *M. tuberculosis* strain via the repression of bacterial growth and the induction of bacterial persistence *in vivo*. Nevertheless, the biological function and signaling pathway of *Rv2629* warrants further investigation.

## Author Contributions

QW and LZ conceived and supervised the study; DL, KH, WW, CP, and YD performed experiments. RJ and WX provided new tools and reagents; HyW and HhW analyzed data and helped to design experiments; DL, QW and LZ wrote the manuscript. All authors reviewed the results and approved the final version of the manuscript.

## Conflict of Interest Statement

The authors declare that the research was conducted in the absence of any commercial or financial relationships that could be construed as a potential conflict of interest.

## Supplementary Material

**FIGURE S1 |** Expression levels of *Rv2629* modulate the growth of *H37Ra* strains under aerobic conditions. The growth curve of *recombinant H37Ra* overexpressing the *Rv2629^W^* (*RaW*) and/or *Rv2629^M^* (*RaM*) genes were measured using the BD BACTEC MGIT 960 *Mycobacterium* growth and detection system. The results are shown as the fluorescence intensity acquired automatically. *RaP* (transformed with the pMV261 plasmid) was used as a control. The results indicated comparatively slow growth of *RaW*. Data are representative of two experiments.

**FIGURE S2 |** The growth of MSL in THP-1 cells infected at different multiplicity of infections (MOI). **(A)** The *M. smegmatis* low-expressing *MSMEG_1130* (*MSL*) strain exhibited a rapid growth in THP-1 cells at 72 and 96 h post-infection, when infected at a MOI value of 1:5, *P* < 0.001(72 and 96 h). **(B)** *M. smegmatis* low-expressing *MSMEG_1130* (*MSL*) strain exhibited a rapid growth in THP-1 cells at 72 and 96 h post-infection, when infected at a MOI value of 1:20, *P* < 0.05 (72 and 96 h). The data of each time point was analyzed by the Student’s *t*-test after homogeneity test of variance, *n* = 4.

## Abbreviations

AA: amino-acid acetytransferase
ACP: acr-coregulated promoter
ADC: albumin-dextrose-catalase
BCIP/NBT: 5-bromo-4-chloro-3-indolylphosphate/nitroblue tetrazolium
CHP: conserved hypothetical protein
FFP: ferritin family protein
FtsZ: filamentous temperature-sensitive protein Z
GO: Gene Ontology
MDR: multidrug resistance
MS: mass spectrometry
SD: standard deviation
TB: tuberculosis
TF: trigger factor
UK: uridylate kinase

## References

[B1] Alonso M. Navarro Y. Barletta F. Martinez Lirola M. Gotuzzo E. Bouza E. (2011). A novel method for the rapid and prospective identification of Beijing *Mycobacterium tuberculosis* strains by high-resolution melting analysis. *Clin. Microbiol. Infect.* 17 349–357. 10.1111/j.1469-0691.2010.03234.x 20384709

[B2] Altaf M. Miller C. H. Bellows D. S. O’Toole R. (2010). Evaluation of the *Mycobacterium smegmatis* and BCG models for the discovery of *Mycobacterium tuberculosis* inhibitors. *Tuberculosis* 90 333–337. 10.1016/j.tube.2010.09.002 20933470

[B3] Andreu N. Soto C. Y. Roca I. Martin C. Gibert I. (2004). *Mycobacterium smegmatis* displays the *Mycobacterium tuberculosis* virulence-related neutral red character when expressing the Rv0577 gene. *FEMS Microbiol. Lett.* 231 283–289. 10.1016/S0378-1097(04)00008-4 14987776

[B4] Bae H. J. Lee H. N. Baek M. N. Park E. J. Eom C. Y. Ko I. J. (2017). Inhibition of the DevSR two-component system by overexpression of *Mycobacterium tuberculosis* PknB in *Mycobacterium smegmatis*. *Mol. Cells* 40 632–642. 10.14348/molcells.2017.0076 28843272 PMC5638771

[B5] Bartek I. L. Rutherford R. Gruppo V. Morton R. A. Morris R. P. Klein M. R. (2009). The DosR regulon of *M. tuberculosis* and antibacterial tolerance. *Tuberculosis* 89 310–316. 10.1016/j.tube.2009.06.001 19577518 PMC2718728

[B6] Belanger A. E. Hatfull G. F. (1999). Exponential-phase glycogen recycling is essential for growth of *Mycobacterium smegmatis*. *J. Bacteriol.* 181 6670–6678. 10542168 10.1128/jb.181.21.6670-6678.1999 PMC94131

[B7] Boon C. Dick T. (2002). Mycobacterium bovis BCG response regulator essential for hypoxic dormancy. *J. Bacteriol.* 184 6760–6767. 10.1128/JB.184.24.6760-6767.2002 12446625 PMC135468

[B8] Caviedes L. Delgado J. Gilman R. H. (2002). Tetrazolium microplate assay as a rapid and inexpensive colorimetric method for determination of antibiotic susceptibility of *Mycobacterium tuberculosis*. *J. Clin. Microbiol.* 40 1873–1874. 10.1128/JCM.40.5.1873-1874.2002 11980982 PMC130930

[B9] Chakravorty S. Aladegbami B. Motiwala A. S. Dai Y. Safi H. Brimacombe M. (2008). Rifampin resistance, Beijing-W clade-single nucleotide polymorphism cluster group 2 phylogeny, and the Rv2629 191-C allele in *Mycobacterium tuberculosis* strains. *J. Clin. Microbiol.* 46 2555–2560. 10.1128/JCM.00666-08 18550732 PMC2519504

[B10] Daugelat S. Kowall J. Mattow J. Bumann D. Winter R. Hurwitz R. (2003). The RD1 proteins of *Mycobacterium tuberculosis*: expression in *Mycobacterium smegmatis* and biochemical characterization. *Microbes Infect.* 5 1082–1095. 10.1016/S1286-4579(03)00205-3 14554249

[B11] Deng W. Long Q. Zeng J. Li P. Yang W. Chen X. (2017). *Mycobacterium tuberculosis* PE_PGRS41 enhances the intracellular survival of *M. smegmatis* within macrophages via blocking innate immunity and inhibition of host defense. *Sci. Rep.* 7:46716. 10.1038/srep46716 28440335 PMC5404228

[B12] Domenech P. Zou J. Averback A. Syed N. Curtis D. Donato S. (2017). Unique regulation of the DosR regulon in the Beijing lineage of *Mycobacterium tuberculosis*. *J. Bacteriol.* 199:e00696–16. 10.1128/JB.00696-16 27799329 PMC5198487

[B13] Doosti-Irani A. Ayubi E. Mostafavi E. (2016). Tuberculin and QuantiFERON-TB-Gold tests for latent tuberculosis: a meta-analysis. *Occup. Med.* 66 437–445. 10.1093/occmed/kqw035 27121635

[B14] Errey J. C. Blanchard J. S. (2005). Functional characterization of a novel ArgA from *Mycobacterium tuberculosis*. *J. Bacteriol.* 187 3039–3044. 10.1128/JB.187.9.3039-3044.2005 15838030 PMC1082834

[B15] Falcone V. Bassey E. Jacobs W. Jr. Collins F. (1995). The immunogenicity of recombinant *Mycobacterium smegmatis* bearing BCG genes. *Microbiology* 141(Pt 5), 1239–1245. 10.1099/13500872-141-5-1239 7773417

[B16] Fassy F. Krebs O. Lowinski M. Ferrari P. Winter J. Collard-Dutilleul V. (2004). UMP kinase from *Streptococcus pneumoniae*: evidence for co-operative ATP binding and allosteric regulation. *Biochem. J.* 384(Pt 3), 619–627. 10.1042/BJ20040440 15324307 PMC1134148

[B17] Florczyk M. A. McCue L. A. Purkayastha A. Currenti E. Wolin M. J. McDonough K. A. (2003). A family of acr-coregulated *Mycobacterium tuberculosis* genes shares a common DNA motif and requires Rv3133c (dosR or devR) for expression. *Infect. Immun.* 71 5332–5343. 10.1128/IAI.71.9.5332-5343.2003 12933881 PMC187371

[B18] Fu L. M. Fu-Liu C. S. (2007). The gene expression data of *Mycobacterium tuberculosis* based on Affymetrix gene chips provide insight into regulatory and hypothetical genes. *BMC Microbiol.* 7:37. 10.1186/1471-2180-7-37 17501996 PMC1884158

[B19] Gharahdaghi F. Weinberg C. R. Meagher D. A. Imai B. S. Mische S. M. (1999). Mass spectrometric identification of proteins from silver-stained polyacrylamide gel: a method for the removal of silver ions to enhance sensitivity. *Electrophoresis* 20 601–605. 10.1002/(SICI)1522-2683(19990301)20:3<601::AID-ELPS601>3.0.CO;2-6 10217175

[B20] Hoffmann A. Bukau B. Kramer G. (2010). Structure and function of the molecular chaperone Trigger Factor. *Biochim. Biophys. Acta* 1803 650–661. 10.1016/j.bbamcr.2010.01.017 20132842

[B21] Homolka S. Koser C. Archer J. Rusch-Gerdes S. Niemann S. (2009). Single-nucleotide polymorphisms in Rv2629 are specific for *Mycobacterium tuberculosis* genotypes Beijing and Ghana but not associated with rifampin resistance. *J. Clin. Microbiol.* 47 223–226. 10.1128/JCM.01237-08 19020060 PMC2620844

[B22] Kassa D. Ran L. Geberemeskel W. Tebeje M. Alemu A. Selase A. (2012). Analysis of immune responses against a wide range of *Mycobacterium tuberculosis* antigens in patients with active pulmonary tuberculosis. *Clin. Vaccine Immunol.* 19 1907–1915. 10.1128/CVI.00482-12 23015647 PMC3535869

[B23] Keshari D. Singh K. S. Sharma R. Yadav S. Singh S. K. (2017). MSMEG_5684 down-regulation in *Mycobacterium smegmatis* affects its permeability, survival under stress and persistence. *Tuberculosis* 103 61–70. 10.1016/j.tube.2017.01.004 28237035

[B24] Kuo C. J. Bell H. Hsieh C. L. Ptak C. P. Chang Y. F. (2012). Novel mycobacteria antigen 85 complex binding motif on fibronectin. *J. Biol. Chem.* 287 1892–1902. 10.1074/jbc.M111.298687 22128161 PMC3265870

[B25] Kwon K. W. Kim W. S. Kim H. Han S. J. Hahn M. Y. Lee J. S. (2017). Novel vaccine potential of Rv3131, a DosR regulon-encoded putative nitroreductase, against hyper-virulent *Mycobacterium tuberculosis* strain K. *Sci. Rep.* 7:44151. 10.1038/srep44151 28272457 PMC5341159

[B26] Lauten E. H. Pulliam B. L. DeRousse J. Bhatta D. Edwards D. A. (2010). Gene expression, bacteria viability and survivability following spray drying of *Mycobacterium smegmatis*. *Materials (Basel)* 3 2684–2724. 10.3390/ma3042684

[B27] Lee S. E. Kim S. Y. Kim C. M. Kim M. K. Kim Y. R. Jeong K. (2007). The pyrH gene of *Vibrio vulnificus* is an essential in vivo survival factor. *Infect. Immun.* 75 2795–2801. 10.1128/IAI.01499-06 17371864 PMC1932866

[B28] Louw G. E. Warren R. M. van Helden P. D. Victor T. C. (2009). Rv2629 191A/C nucleotide change is not associated with rifampicin resistance in *Mycobacterium tuberculosis*. *Clin. Chem. Lab. Med.* 47 500–501. 10.1515/CCLM.2009.111 19327127

[B29] Mohn W. W. van der Geize R. Stewart G. R. Okamoto S. Liu J. Dijkhuizen L. (2008). The actinobacterial mce4 locus encodes a steroid transporter. *J. Biol. Chem.* 283 35368–35374. 10.1074/jbc.M805496200 18955493 PMC5218832

[B30] Niki M. Niki M. Tateishi Y. Ozeki Y. Kirikae T. Lewin A. (2012). A novel mechanism of growth phase-dependent tolerance to isoniazid in mycobacteria. *J. Biol. Chem.* 287 27743–27752. 10.1074/jbc.M111.333385 22648414 PMC3431685

[B31] Pandey A. K. Sassetti C. M. (2008). Mycobacterial persistence requires the utilization of host cholesterol. *Proc. Natl. Acad. Sci. U.S.A.* 105 4376–4380. 10.1073/pnas.0711159105 18334639 PMC2393810

[B32] Park H. D. Guinn K. M. Harrell M. I. Liao R. Voskuil M. I. Tompa M. (2003). Rv3133c/dosR is a transcription factor that mediates the hypoxic response of *Mycobacterium tuberculosis*. *Mol. Microbiol.* 48 833–843. 10.1046/j.1365-2958.2003.03474.x 12694625 PMC1992516

[B33] Peddireddy V. Doddam S. N. Ahmed N. (2017). Mycobacterial dormancy systems and host responses in tuberculosis. *Front. Immunol.* 8:84. 10.3389/fimmu.2017.00084 28261197 PMC5309233

[B34] Perkowski E. F. Miller B. K. McCann J. R. Sullivan J. T. Malik S. Allen I. C. (2016). An orphaned Mce-associated membrane protein of *Mycobacterium tuberculosis* is a virulence factor that stabilizes Mce transporters. *Mol. Microbiol.* 100 90–107. 10.1111/mmi.13303 26712165 PMC5028898

[B35] Ramaswamy S. V. Reich R. Dou S. J. Jasperse L. Pan X. Wanger A. (2003). Single nucleotide polymorphisms in genes associated with isoniazid resistance in *Mycobacterium tuberculosis*. *Antimicrob. Agents Chemother.* 47 1241–1250. 10.1128/AAC.47.4.1241-1250.2003 12654653 PMC152487

[B36] Reed M. B. Gagneux S. Deriemer K. Small P. M. Barry C. E. III. (2007). The W-Beijing lineage of *Mycobacterium tuberculosis* overproduces triglycerides and has the DosR dormancy regulon constitutively upregulated. *J. Bacteriol.* 189 2583–2589. 10.1128/JB.01670-06 17237171 PMC1855800

[B37] Rodriguez D. C. Ocampo M. Varela Y. Curtidor H. Patarroyo M. A. Patarroyo M. E. (2015). Mce4F *Mycobacterium tuberculosis* protein peptides can inhibit invasion of human cell lines. *Pathog. Dis.* 73:ftu020. 10.1093/femspd/ftu020 25743470

[B38] Rostirolla D. C. Breda A. Rosado L. A. Palma M. S. Basso L. A. Santos D. S. (2011). UMP kinase from *Mycobacterium tuberculosis*: mode of action and allosteric interactions, and their likely role in pyrimidine metabolism regulation. *Arch. Biochem. Biophys.* 505 202–212. 10.1016/j.abb.2010.10.019 21035424

[B39] Scheler C. Lamer S. Pan Z. Li X. P. Salnikow J. Jungblut P. (1998). Peptide mass fingerprint sequence coverage from differently stained proteins on two-dimensional electrophoresis patterns by matrix assisted laser desorption/ionization-mass spectrometry (MALDI-MS). *Electrophoresis* 19 918–927. 10.1002/elps.1150190607 9638938

[B40] Starck J. Kallenius G. Marklund B. I. Andersson D. I. Akerlund T. (2004). Comparative proteome analysis of *Mycobacterium tuberculosis* grown under aerobic and anaerobic conditions. *Microbiology* 150(Pt 11), 3821–3829. 10.1099/mic.0.27284-0 15528667

[B41] Sun C. Yang G. Yuan J. Peng X. Zhang C. Zhai X. (2017). *Mycobacterium tuberculosis* hypoxic response protein 1 (Hrp1) augments the pro-inflammatory response and enhances the survival of *Mycobacterium smegmatis* in murine macrophages. *J. Med. Microbiol.* 66 1033–1044. 10.1099/jmm.0.000511 28671529

[B42] Sweeney K. A. Dao D. N. Goldberg M. F. Hsu T. Venkataswamy M. M. Henao-Tamayo M. (2011). A recombinant *Mycobacterium smegmatis* induces potent bactericidal immunity against *Mycobacterium tuberculosis*. *Nat. Med.* 17 1261–1268. 10.1038/nm.2420 21892180 PMC3250071

[B43] Trauner A. Lougheed K. E. Bennett M. H. Hingley-Wilson S. M. Williams H. D. (2012). The dormancy regulator DosR controls ribosome stability in hypoxic mycobacteria. *J. Biol. Chem.* 287 24053–24063. 10.1074/jbc.M112.364851 22544737 PMC3390679

[B44] Tumminia S. J. Mandiyan V. Wall J. S. Boublik M. (1991). Heterogeneity of *Escherichia coli* ribosomes established by scanning transmission electron microscopy. *Biochimie* 73 919–925. 10.1016/0300-9084(91)90133-L 1742364

[B45] Voskuil M. I. Schnappinger D. Visconti K. C. Harrell M. I. Dolganov G. M. Sherman D. R. (2003). Inhibition of respiration by nitric oxide induces a *Mycobacterium tuberculosis* dormancy program. *J. Exp. Med.* 198 705–713. 10.1084/jem.20030205 12953092 PMC2194188

[B46] Wang L. Zuo M. Chen H. Liu S. Wu X. Cui Z. (2017). *Mycobacterium tuberculosis* Lipoprotein MPT83 induces apoptosis of infected macrophages by activating the TLR2/p38/COX-2 signaling pathway. *J. Immunol.* 198 4772–4780. 10.4049/jimmunol.1700030 28507027

[B47] Wang Q. Yue J. Zhang L. Xu Y. Chen J. Zhang M. (2007). A newly identified 191A/C mutation in the Rv2629 gene that was significantly associated with rifampin resistance in *Mycobacterium tuberculosis*. *J. Proteome Res.* 6 4564–4571. 10.1021/pr070242z 17970586

[B48] Wu T. Zhao Z. Zhang L. Ma H. Lu K. Ren W. (2011). Trigger factor of *Streptococcus suis* is involved in stress tolerance and virulence. *Microb. Pathog.* 51 69–76. 10.1016/j.micpath.2010.10.001 21093574

[B49] Yamanaka K. Ogura T. Niki H. Hiraga S. (1992). Identification and characterization of the smbA gene, a suppressor of the mukB null mutant of *Escherichia coli*. *J. Bacteriol.* 174 7517–7526. 10.1128/jb.174.23.7517-7526.1992 1447125 PMC207461

[B50] Yoshida T. Nasu H. Namba E. Ubukata O. Yamashita M. (2012). Discovery of a compound which acts as a bacterial UMP kinase PyrH inhibitor. *FEMS Microbiol. Lett.* 330 121–126. 10.1111/j.1574-6968.2012.02546.x 22428584

[B51] Yu Z. Zhang C. Zhou M. Li Q. Li H. Duan W. (2017). *Mycobacterium tuberculosis* PPE44 (Rv2770c) is involved in response to multiple stresses and promotes the macrophage expression of IL-12 p40 and IL-6 via the p38, ERK, and NF-kappaB signaling axis. *Int. Immunopharmacol.* 50 319–329. 10.1016/j.intimp.2017.06.028 28743081

[B52] Zhang L. Xu W. Cui Z. Liu Y. Wang W. Wang J. (2014). A novel method of identifying *Mycobacterium tuberculosis* Beijing strains by detecting SNPs in Rv0444c and Rv2629. *Curr. Microbiol.* 68 381–386. 10.1007/s00284-013-0487-2 24218231

[B53] Zimhony O. Vilcheze C. Jacobs W. R. Jr. (2004). Characterization of *Mycobacterium smegmatis* expressing the *Mycobacterium tuberculosis* fatty acid synthase I (fas1) gene. *J. Bacteriol.* 186 4051–4055. 10.1128/JB.186.13.4051-4055.2004 15205406 PMC421601

