# Supplementary figures and images for: Rv2629 Overexpression Delays Mycobacterium smegmatis and Mycobacteria tuberculosis Entry into Log-Phase and Increases Pathogenicity of Mycobacterium smegmatis in Mice
Source: Front Microbiol. 2017 Nov 15;8:2231. doi: 10.3389/fmicb.2017.02231 (PMC5694894; doi:10.3389/fmicb.2017.02231)

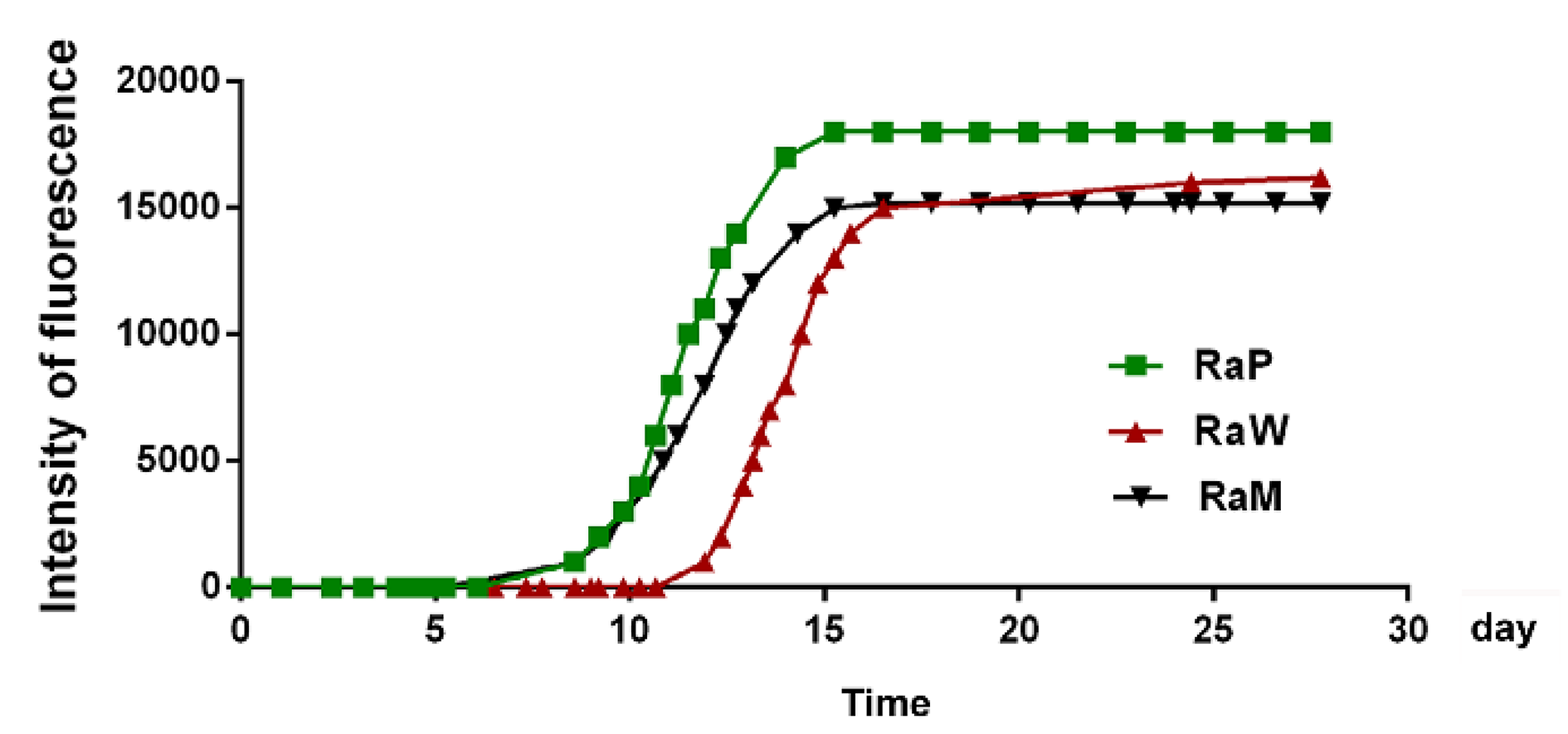

Supplement: Supplementary file 1 [file Image_1.TIF]

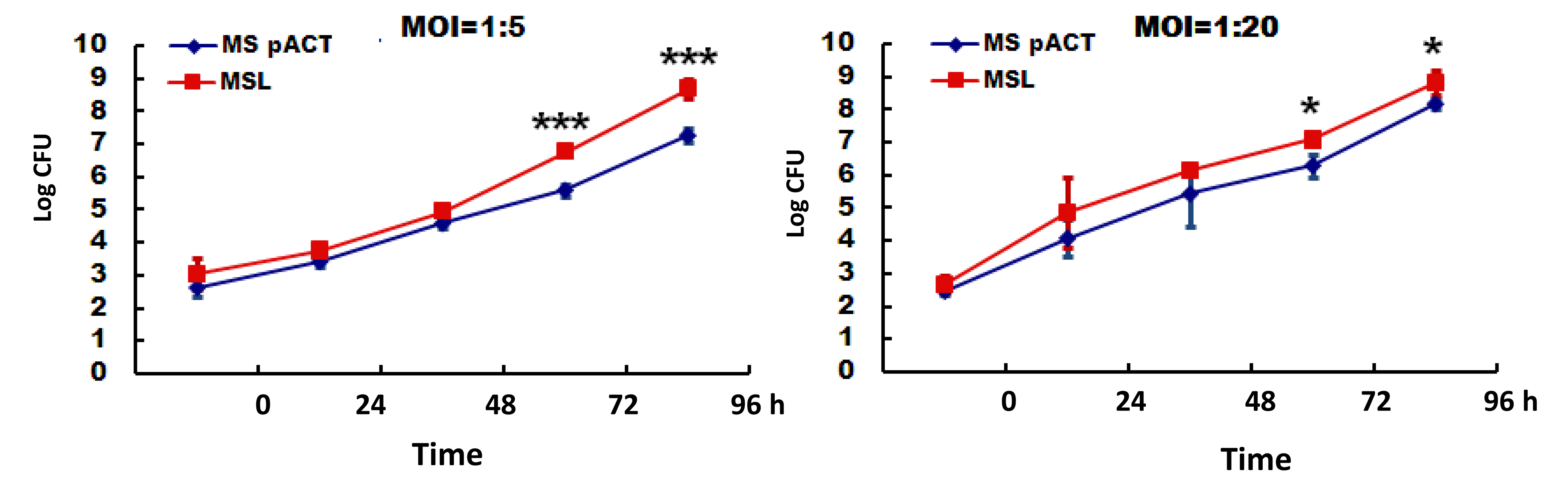

Supplement: Supplementary file 2 [file Image_2.TIF]
