# Supplementary material for: Rv2629 Overexpression Delays Mycobacterium smegmatis and Mycobacteria tuberculosis Entry into Log-Phase and Increases Pathogenicity of Mycobacterium smegmatis in Mice
Source: Front Microbiol. 2017 Nov 15;8:2231. doi: 10.3389/fmicb.2017.02231 (PMC5694894; doi:10.3389/fmicb.2017.02231)
Supplement: Supplementary file 3 [file Table_1.DOC]

**Table S1. Primers used in this study**

| **Primers for cloning mycobacterial shuttle vector into *E. coli*** | | | | |
| --- | --- | --- | --- | --- |
| **Gene** | **Vector** | **Primer** | **Description** | **Primer sequence (5′-3′)** |
| Rv2629 | pMV261 | Forward | *Bam*H I | TA**GGATCC**ATGCGATCAGAACGTCTCCG |
|  |  | Reverse | *Hind*III | GAT**AAGCTT**CTAGGATCTATGGCTGCCGAGT |
| MSMEG_1130 | pACT | Forward | *Eco*RI | CA**GAATTC**GTGGACGTTTTGGAAC |
|  |  | Reverse | *Bam*H I | AT**GGATCC**TCACCAGCGCAACAC |
| **Primers for RT-PCR** | | | | |
| **Gene** | **Sites** | **Primer** | **Primer sequence (5′-3′)** | |
| Rv2629 | 616–638 bp | Forward | 5′-ACAGACCTGCTTTCCACATTGCC-3′ | |
|  | 777–797 bp | Reverse | 5′-CGTCCGATCTCCGCCTCAAAT-3′ | |
| 16S rRNA | 509–528 bp | Forward | 5′-GTAGGGTGCGAGCGTTGTCC-3′ | |
|  | 728–749 bp | Reverse | 5′-CAGCGTCAGTTACTGCCCAGAG-3′ | |
| *Rv2703* | 594–613 bp | Forward | 5′-CTCCGGTGATTTCGTCTGGG-3′ | |
|  | 812–828 bp | Reverse | 5′-GGCGGCAGGCAGCTTTT-3′ | |
