# Supplementary material for: Rv2629 Overexpression Delays Mycobacterium smegmatis and Mycobacteria tuberculosis Entry into Log-Phase and Increases Pathogenicity of Mycobacterium smegmatis in Mice
Source: Front Microbiol. 2017 Nov 15;8:2231. doi: 10.3389/fmicb.2017.02231 (PMC5694894; doi:10.3389/fmicb.2017.02231)
Supplement: Supplementary file 4 [file Table_2.doc]

**Table S2. Types of strains used with the corresponding genes and plasmids for overexpression and reduced expression of Rv2629**

| Type of strain | Abbreviation | Gene | Plasmid |
| --- | --- | --- | --- |
| *M. smegmatis MC2 155* | MSW (wild type) | Rv2629 | pMV261 (overexpressing) |
| *M. smegmatis MC2 155* | MSM (mutant) | Rv2629 191C mutation | pMV261 (overexpressing) |
| *M. tuberculosis H37Ra* | RaW (wild type) | Rv2629 | pMV261 (overexpressing) |
| *M. tuberculosis H37Ra* | RaM (mutant) | Rv2629 191C mutation | pMV261 (overexpressing) |
| *M. smegmatis MC2 155* | MSL | MSMEG_1130 knock down | pACT  (low expressing) |
| *M. smegmatis MC2 155* | MSP | Empty vector | pMV261 (control) |
| *M. smegmatis MC2 155* | MSpACT | Empty vector | pACT (control) |
| *M. tuberculosis H37Ra* | RaP | Empty vector | pMV261 (control) |
