# Supplementary material for: Rv2629 Overexpression Delays Mycobacterium smegmatis and Mycobacteria tuberculosis Entry into Log-Phase and Increases Pathogenicity of Mycobacterium smegmatis in Mice
Source: Front Microbiol. 2017 Nov 15;8:2231. doi: 10.3389/fmicb.2017.02231 (PMC5694894; doi:10.3389/fmicb.2017.02231)
Supplement: Supplementary file 5 [file Table_3.DOC]

**Table S3. 122 genes correlated with wild type Rv2629**

| **ProbeName** | **Gene** | **Product Annotations** | **RaW Vs Contrl** | **RaM Vs Control** | **Raw Vs RaM** |
| --- | --- | --- | --- | --- | --- |
| MRA_3860 | papA2 | putative polyketide synthase associated protein PapA2 | up | up | up |
| MRA_3766 |  | hypothetical protein | up | up | up |
| MRA_3684 |  | DeaD/DeaH box family ATP-dependent RNA helicase | up | up | up |
| MRA_3551 |  | PE-PGRS family protein | up | up | up |
| MRA_3537 | mce4C | MCE-family protein Mce4C | up | down | up |
| MRA_3532 |  | Mce associated protein | up | up | up |
| MRA_3487 |  | hypothetical protein | up | up | up |
| MRA_3427 |  | putative transposase | up | up | down |
| MRA_3405 |  | hypothetical protein | up | up | up |
| MRA_3403 |  | hypothetical protein | up | up | up |
| MRA_3402 |  | putative ATP/GTP-binding protein | up | up | up |
| MRA_3356 | deoA | thymidine phosphorylase | up | up | up |
| MRA_3355 | add | adenosine deaminase | up | down | up |
| MRA_3347 | amiB1 | putative amidase AmiB1 | up | up | up |
| MRA_3280 |  | putative integral membrane transport protein | down | down | up |
| MRA_3189 | nuoL | NADH dehydrogenase subunit L | up | up | up |
| MRA_3186 | nuoI | NADH dehydrogenase subunit I | up | up | up |
| MRA_3184 | nuoG | NADH dehydrogenase subunit G | up | up | up |
| MRA_3182 | nuoE | NADH dehydrogenase subunit E | up | up | up |
| MRA_3180 | nuoC | NADH dehydrogenase subunit C | up | up | up |
| MRA_3144 | moaD1 | molybdenum cofactor biosynthesis protein D1 | up | down | up |
| MRA_3063 |  | glycosyl transferase | up | up | up |
| MRA_3031 | ilvC | ketol-acid reductoisomerase | up | up | up |
| MRA_2977 | fadD29 | acyl-CoA synthetase | up | up | up |
| MRA_2975 | fadD22 | acyl-CoA synthetase | up | up | up |
| MRA_2973 | pks1 | putative polyketide synthase Pks1 | up | up | up |
| MRA_2960 | ppsD | phenolpthiocerol synthesis type-I polyketide synthase PpsD | up | up | up |
| MRA_2936 | dacB2 | putative D-alanyl-D-alanine carboxypeptidase DacB2 | up | down | up |
| MRA_2927 | rnhB | ribonuclease HII | up | up | up |
| MRA_2846 |  | hypothetical protein | up | up | down |
| MRA_2807 | gpsI | polynucleotide phosphorylase/polyadenylase | up | up | up |
| MRA_2661 |  | hypothetical protein | up | up | up |
| MRA_2654 |  | hypothetical protein | up | down | up |
| MRA_2652 |  | hypothetical protein | up | down | up |
| MRA_2632 |  | glutamine amidotransferase subunit PdxT | up | up | up |
| MRA_2629 | speE | spermidine synthase | up | up | up |
| MRA_2570 |  | hypothetical protein | up | up | up |
| MRA_2510 | lipQ | carboxylesterase LipQ | up | down | up |
| MRA_2427 | lppR | putative lipoprotein LppR | up | up | up |
| MRA_2412 | hemN | coproporphyrinogen III oxidase | up | up | up |
| MRA_2388 | era | GTP-binding protein Era | down | up | down |
| MRA_2339 |  | hypothetical protein | up | up | down |
| MRA_2288 | cyp128 | putative cytochrome p450 128 CYP128 | up | up | up |
| MRA_2273 |  | putative secreted protein | up | up | up |
| MRA_2250 | cobC | hypothetical protein | up | up | up |
| MRA_2237 | glnA1 | glutamine synthetase | up | up | up |
| MRA_2216 | ctaC | cytochrome c oxidase subunit II | up | up | up |
| MRA_2205 |  | hypothetical protein | up | down | up |
| MRA_2171 | mraY | phospho-N-acetylmuramoyl-pentapeptide- transferase | up | up | up |
| MRA_2128 |  | putative integral membrane protein | up | down | up |
| MRA_2116 |  | hypothetical protein | up | down | up |
| MRA_2076 |  | hypothetical protein | up | up | up |
| MRA_2053 |  | sugar ABC transporter ATP-binding protein | up | up | down |
| MRA_2047 | acg | hypothetical protein | up | down | up |
| MRA_2045 |  | hypothetical protein | up | down | up |
| MRA_2044 | pfkB | phosphofructokinase PfkB | up | down | up |
| MRA_2043 |  | hypothetical protein | up | down | up |
| MRA_2042 |  | histidine kinase response regulator | up | up | up |
| MRA_1948 | ephB | epoxide hydrolase EphB | up | up | down |
| MRA_1944 | fadE17 | acyl-CoA dehydrogenase FadE17 | up | up | down |
| MRA_1897 | fbpB | secreted antigen 85-B FbpB | up | down | up |
| MRA_1896 |  | chorismate mutase | up | down | up |
| MRA_1894 |  | hypothetical protein | up | up | up |
| MRA_1828 |  | putative transcriptional regulatory protein | up | up | up |
| MRA_1727 |  | hypothetical protein | up | up | up |
| MRA_1706 |  | hypothetical protein | up | up | up |
| MRA_1674 | pks17 | putative polyketide synthase Pks17 | up | up | down |
| MRA_1672 | pks7 | putative polyketide synthase Pks7 | up | up | up |
| MRA_1665 | argB | acetylglutamate kinase | up | down | up |
| MRA_1653 | rpmI | 50S ribosomal protein L35 | up | up | up |
| MRA_1644 |  | putative drug transporter | up | down | up |
| MRA_1641 | coaE | dephospho-CoA kinase/unknown domain fusion protein | up | down | up |
| MRA_1624 | lgt | prolipoprotein diacylglyceryl transferase | up | up | up |
| MRA_1623 | trpA | tryptophan synthase subunit alpha | up | up | up |
| MRA_1622 | trpB | tryptophan synthase subunit beta | up | up | up |
| MRA_1582 | bioD | dithiobiotin synthetase | up | up | up |
| MRA_1574 | treZ | maltooligosyl trehalose trehalohydrolase | up | up | up |
| MRA_1564 | frdA | fumarate reductase flavoprotein subunit | up | down | up |
| MRA_1466 |  | antibiotic ABC transporter permease | up | up | up |
| MRA_1461 |  | PE-PGRS family protein | up | up | up |
| MRA_1323 | murA | UDP-N-acetylglucosamine 1-carboxyvinyltransferase | up | up | up |
| MRA_1320 |  | hypothetical protein | up | up | up |
| MRA_1289 | oppD | peptide ABC transporter ATP-binding protein | up | up | up |
| MRA_1132 | bpoB | putative peroxidase BpoB | up | down | up |
| MRA_1001 | moeA1 | molybdopterin biosynthesis protein MoeA1 | up | up | up |
| MRA_0827 |  | hypothetical protein | up | up | up |
| MRA_0787 | cyp126 | putative cytochrome p450 126 CYP126 | up | up | up |
| MRA_0742 | mapA | methionine aminopeptidase | up | down | up |
| MRA_0739 |  | hypothetical protein | up | up | up |
| MRA_0730 | rpmD | 50S ribosomal protein L30 | up | up | up |
| MRA_0724 | rplE | 50S ribosomal protein L5 | up | down | up |
| MRA_0716 | rplP | 50S ribosomal protein L16 | up | up | up |
| MRA_0710 | rplD | 50S ribosomal protein L4 | up | up | up |
| MRA_0705 |  | putative dehydrogenase | up | up | up |
| MRA_0702 | lldD1 | FMN-dependent alpha-hydroxy acid dehydrogenase family protein | up | up | up |
| MRA_0666 | mkl | ribonucleotide ABC transporter ATP-binding protein Mkl | up | up | up |
| MRA_0663 | rplL | 50S ribosomal protein L7/L12 | up | down | up |
| MRA_0639 | recB | exodeoxyribonuclease V subunit beta | up | up | up |
| MRA_0619 |  | hypothetical protein | up | up | up |
| MRA_0548 |  | putative integral membrane protein | up | up | up |
| MRA_0521 |  | putative transmembrane protein | up | up | up |
| MRA_0520 |  | putative transmembrane protein | up | up | up |
| MRA_0517 | hemC | porphobilinogen deaminase | up | up | up |
| MRA_0411 | pks6 | membrane bound polyketide synthase Pks6 | up | up | up |
| MRA_0329 |  | hypothetical protein | up | down | up |
| MRA_0301 |  | putative transmembrane protein | up | down | up |
| MRA_0299 |  | putative transmembrane protein | up | up | up |
| MRA_0298 |  | hypothetical protein | up | up | up |
| MRA_0244 |  | putative transmembrane protein | up | down | up |
| MRA_0234 |  | putative transmembrane protein | up | down | up |
| MRA_0178 | mce1B | MCE-family protein Mce1B | up | up | up |
| MRA_0174 | fadD5 | acyl-CoA synthetase | up | up | up |
| MRA_0165 | pntB | NAD(P) transhydrogenase subunit beta | up | down | up |
| MRA_0150 |  | chloride channel | up | up | up |
| MRA_0146 |  | dihydroflavonol 4-reductase-related protein | up | up | up |
| MRA_0121 | hddA | D-alpha-D-heptose-7-phosphate kinase HddA | up | up | up |
| MRA_0104 |  | hypothetical protein | up | down | up |
| MRA_0090 | hycQ | putative hydrogenase HycQ | up | up | up |
| MRA_0089 | hycP | putative hydrogenase HycP | up | up | up |
| MRA_0088 | hycD | formate hydrogenlyase HycD | up | down | up |
| MRA_0087 |  | putative NADH-ubiquinone oxidoreductase | up | up | up |
| MRA_0085 |  | putative transcriptional regulatory protein | up | down | up |
| MRA_0056 | rpsF | 30S ribosomal protein S6 | up | up | up |
| MRA_0017 | pknA | serine/threonine protein kinase | up | up | up |
