# Supplementary material for: Rv2629 Overexpression Delays Mycobacterium smegmatis and Mycobacteria tuberculosis Entry into Log-Phase and Increases Pathogenicity of Mycobacterium smegmatis in Mice
Source: Front Microbiol. 2017 Nov 15;8:2231. doi: 10.3389/fmicb.2017.02231 (PMC5694894; doi:10.3389/fmicb.2017.02231)
Supplement: Supplementary file 6 [file Table_4.DOC]

**Table S4. Pathway analysis for genes that were correlated with Rv2629 expression**

| Pathway ID | Definition | Fisher P-value |
| --- | --- | --- |
| mra03010 | Ribosome - *Mycobacterium tuberculosis* H37Ra | 4.28468E-10 |
| mra00623 | Toluene degradation - *Mycobacterium tuberculosis* H37Ra | 0.01194141 |
| mra00330 | Arginine and proline metabolism - *Mycobacterium tuberculosis* H37Ra | 0.01729974 |
| mra00190 | Oxidative phosphorylation - *Mycobacterium tuberculosis* H37Ra | 0.02834651 |
| mra03440 | Homologous recombination - *Mycobacterium tuberculosis* H37Ra | 0.03502025 |
